# Protective Effect of Grape Seed Proanthocyanidins on Oxidative Damage of Chicken Follicular Granulosa Cells by Inhibiting FoxO1-Mediated Autophagy

**DOI:** 10.3389/fcell.2022.762228

**Published:** 2022-02-15

**Authors:** Shuo Zhou, An Zhao, Yangyang Wu, Yuling Mi, Caiqiao Zhang

**Affiliations:** College of Animal Sciences, Zhejiang University, Hangzhou, China

**Keywords:** autophagy, grape seed proanthocyanidins, granulosa cell (GC), FoxO1, chicken

## Abstract

A significant decrease in poultry egg production occurs due to ovarian aging and autophagy is one of the important factors of ovarian aging that is induced predominantly by oxidative stress. Increasing evidence showed potential roles of plant-derived grape seed proanthocyanidin (GSPs) in protecting ovarian granulosa cells (GCs) from oxidative damage, although the underlying mechanism is still unclear. Here we investigated the possible functions of autophagy involved in the preventive effect of GSPs on oxidative stress in the GCs of ovarian hierarchical follicles of laying chickens. The results showed that increased autophagy was observed in the aging hens (580-day-old, D580) compared with the peak-lay hens (D280). Treatment of GSPs significantly restored the elevated autophagy and decreased viability of cultured D280 chicken GCs that were elicited by hydrogen peroxide. GSPs also suppressed the increased autophagy in the natural aging hens. Similar to the effect of GSPs on GC viability, inhibition of autophagy also showed a protective effect on the decreased viability of GCs under oxidative damage. However, GSPs were not able to provide further protection in GCs that were pretreated with 3-methyladenine (an autophagy inhibitor). In addition to its promoting action on antioxidant capacity, treatment with GSPs increased survival of GCs from autophagy that was caused by oxidative stress through the FoxO1-related pathway. Inhibition of FoxO1 or activation of PI3K-Akt pathway by GSPs increased the confrontation of GCs to oxidative damage and decreased autophagy in GCs. In addition, activation of the SIRT1 signal inhibited the GCs autophagy that was caused by oxidative stress via GSPs-induced deacetylation of FoxO1. These results revealed a new mechanism of GSPs against oxidative stress of GCs via inhibiting FoxO1, which was probably a possible target for alleviating ovarian aging in laying poultry.

**GRAPHICAL ABSTRACT:**
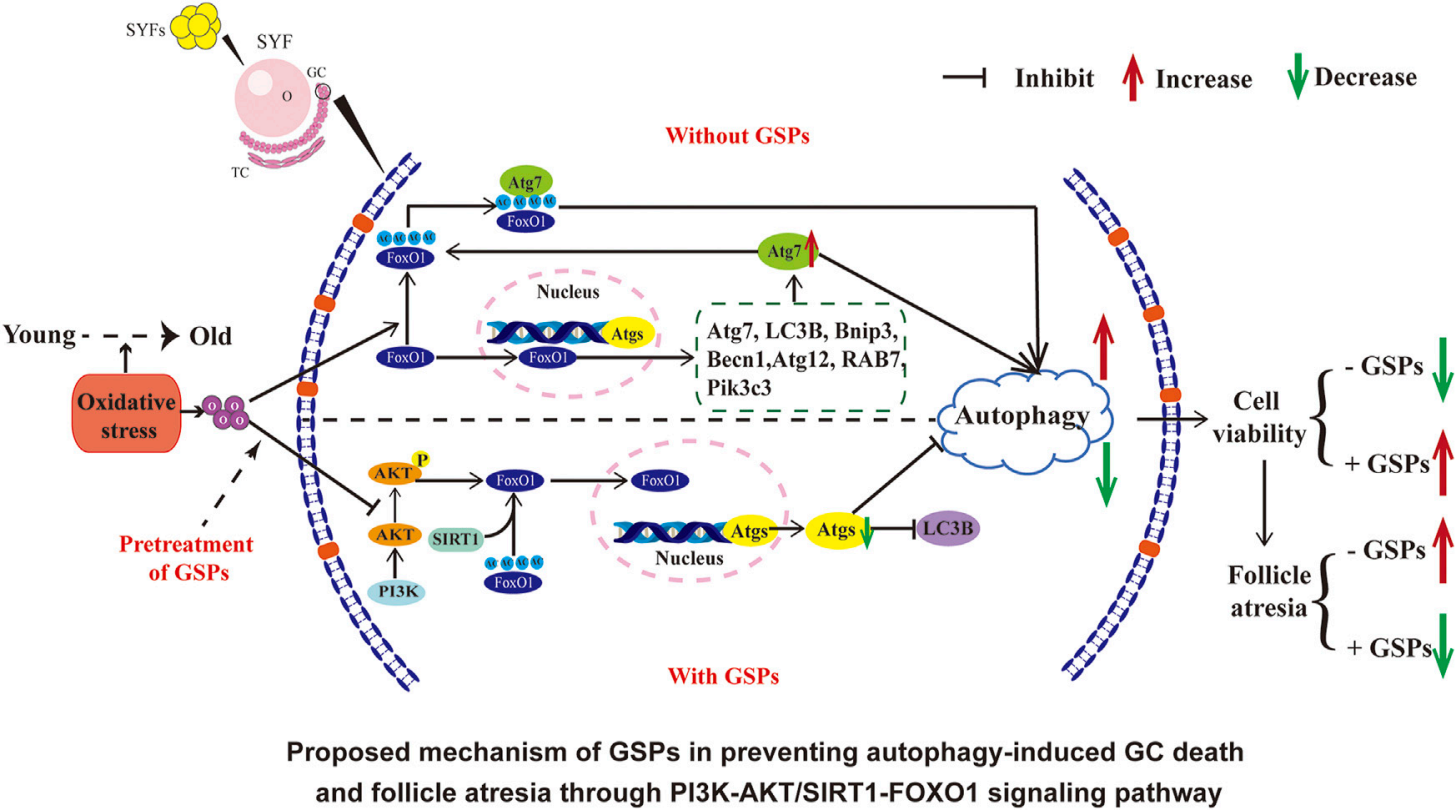


## 1 Introduction

The egg production of the commercial laying chicken decreases around 580 days (D580) and this decrease seriously affects the economic output of laying poultry. Ovarian follicles represent the most important functional unit for the continuation of the avian species and egg production. There are increasing atretic follicles in the aging hens and follicular atresia in poultry is similar to that in mammals, which is mainly due to apoptosis of granulosa cells (GCs) (Matsuda-Minehata et al., 2006). Oxidative stress, which is caused by the accumulation of reactive oxygen species (ROS) in metabolic activities, signifies one of the most important factors leading to ovarian aging (Devine et al., 2001; Luderer, 2014; Lim et al., 2015). Ovarian GCs are highly prone to oxidative damage by ROS attack. Therefore, elucidation of the preventive mechanism of GC death caused by oxidative stress may provide a potential treatment strategy for reproductive failure caused by excessive follicular atresia.

Follicular atresia can be induced by increased cell apoptosis and autophagy (Shen et al., 2017). Autophagy is a lysosomal degradation pathway, which is important for survival, differentiation, development and homeostasis. At the same time, autophagy seems to promote cell death and disease progression (Tan et al., 2016). ROS accelerates autophagy in various manners such as inhibition of the mTOR pathway (Rodrigues et al., 2020). On the contrary, inhibition of ROS production relieved the inhibition of the PI3K/Akt/mTOR pathway to decrease autophagy level (Wang et al., 2018). Elevated autophagy induced apoptosis of rat granulosa cells by decreasing the expression of Bcl-2 and activation of caspase (Choi et al., 2011).

Meanwhile, the Forkhead-box (Fox) protein family is a kind of transcription factor with the wing helix structure in the DNA binding region. Fox is widely involved in physiological processes such as autophagy, cell apoptosis, proliferation, DNA damage repair, differentiation, cell cycle arrest, stress response, aging, metabolism (Katoh and Katoh, 2004). Recent studies show that FoxO1 also regulates the development of follicles and atresia (Zhang et al., 2017; Shen et al., 2017; Liu X. et al., 2018). Immunohistochemical analysis manifested that FoxO1 was localized in the nuclei of GCs of rat atresia follicles (Shen et al., 2012). In the mouse, FoxO1 induced GCs apoptosis and follicles atresia by inhibiting cell proliferation and steroid hormone synthesis (Shen et al., 2017). Though the previous studies have proven that FoxO1 was induced by oxidative stress, the role of FoxO1 in GCs apoptosis and follicular atresia is rarely investigated.

Silent information regulator of transcription 1 (SIRT1), a member of the family of silent transcriptional regulators, has the role of deacetylase and plays an important role in many biological processes, including oxidative stress, apoptosis and senescence, gene transcription, metabolism and so on (Alves-Fernandes and Jasiulionis, 2019; Chen et al., 2020). It was reported that SIRT1 activated FoxO1 through deacetylation and reduced cell oxidative stress injury caused by H_2_O_2_ resulting in osteoblast apoptosis inhibition (Yao et al., 2018). Lin et al. showed that SIRT1 deacetylated p53 and inhibit p53 activation, thereby protecting renal tubular cells from oxidative stress damage and reducing cell apoptosis. Therefore, SIRT1 inhibited p53 activation through deacetylation, reducing cell apoptosis caused by oxidative stress damage (Lin X. et al., 2018). However, the regulatory mechanisms of SIRT1 protein in autophagy during chicken aging remain unclear.

Antioxidants targeting autophagy may contribute to the survival of the GCs against oxidative damage. In poultry production, it’s extremely important to develop attenuating measures to effectively prevent advanced ovarian aging to extend the laying period. Numerous plant-derived natural compounds or synthesized chemicals have been evaluated for their effects in preventing ovarian aging, thus enhancing the poultry laying performance (Yao et al., 2020). In recent years, some natural compounds with antioxidant capacities, such as melatonin (Ming et al., 2018), lycopene (Liu X. et al., 2018), and aloe-emodin (Lu et al., 2007), have been widely studied to postpone aging. The most widely-used proanthocyanidins, grape seed proanthocyanidins (GSPs), is a kind of biological flavonoid with a special molecular structure that possesses diverse functions such as anti-oxidation, anti-allergy, anti-aging, and improving immunity (Shi, 2003; Li et al., 2020; Rigotti et al., 2020; Fu et al., 2021). GSPs improved the antioxidant capacity of the chicken and alleviated the oxidative stress caused by coccidia (Wang et al., 2008). GSPs also enhanced the antioxidant capacity of the aging ovarian tissue, maintained the balance of ovarian cell proliferation and apoptosis and alleviated the decline of the aging ovary (Liu X. T. et al., 2018).

Our previous studies have shown that GSPs treatment can efficiently reduce oxidative stress *via* preventing the ovarian aging process in chickens (Liu X. T. et al., 2018). Here we further explored the mechanism of GSPs in preventing autophagy-induced GCs death and follicle atresia by investigating the PI3K-AKT/SIRT1-FoxO1 signaling pathway. Our findings may provide new understandings of the mechanism of GSPs involved in resistance to ovarian aging in senescent hens.

## 2 Materials and Methods

### 2.1 Animals and Ethics

All procedures were performed in accordance with the Guiding Principles for the Care and Use of Laboratory Animals of Zhejiang University (ZJU20170660). Hyline white hens (Gallus domesticus) were purchased from the farm and fed for free. Sample collection was performed from 280 ± 20 days (D280, high-laying hens) and 580 ± 20 days (D580, aged hens with low-laying). Ovaries were obtained under sterile conditions and extra tissues were removed using fine tweezers and scalpels. Prehierarchical small white follicles (SWFs) were collected for subsequent H&E staining, immunohistochemical staining, and Western blot analysis. The experimental protocols were approved by the Committee on the Ethics of Animal Experiments of Zhejiang University (No. ZJU2015-156-12).

### 2.2 Culture and Treatment of SWFs

The SWFs from D580 hens were transferred to the DMEM high glucose (Hyclone, Tauranga, New Zealand) supplemented with 5% fetal calf serum (FCS; Hyclone, Utah), 100 IU/ml penicillin (Beyotime Biotechnology, Shanghai, China), 100 mg/ml streptomycin (Hyclone, Fremont, CA, United States), 2 mM glutamine and insulin-transferrin-selenium mixture (ITS: 10 mg/ml insulin, 5 mg/ml transferrin and 30 nM selenite). The follicles were cultured in 48-well culture plates (Corning Inc., Corning) with the medium at 38.5°C with 5% CO_2_ for 72 h (Zhou et al., 2021). The medium was renewed every 24 h. The cultured SWFs from D580 hens were treated with 5 μg/ml (8.5 μM) GSPs (Liu X. T. et al., 2018) or/and 10 mM 3-methyladenine (3-MA).

### 2.3 Cell Culture and Treatments

Small yellow follicles (SYFs, 6-8 mm) were removed from D280 or D580 hens and transferred to M199 medium (Hyclone, Tauranga, New Zealand). Granulosa layers (GLs) were separated from the SYFs after washing with ice-cold phosphate-buffered saline (PBS) three times. The GLs were washed several times to remove the attached yolk in cold M199 medium and then was digested with 1 mg/ml collagenase II (Gibco, Grand Island, NY) for 3 min at 37°C. The dispersed GCs were filtered through a 200 μM mesh and the dispersed GCs were centrifuged for 8 min at 1200 rpm. The precipitate was washed three times with ice-cold DMEM. Cell number and survival rate were estimated with trypan blue exclusion test. The cells were seeded at a density of 10^5^ cells/well in collagen-coated 96-well plates with 500 μL DMEM/well supplemented with 1 × ITS, 10% FCS, 100 IU/ml penicillin and 100 μg/ml streptomycin. Cells were cultured at 38.5°C with 5% CO_2_.

#### 2.3.1 Experiment 1: Establishment of the Oxidative Damage Model

For senescence induction, a modified H_2_O_2_ treatment protocol was used (Yao et al., 2020). Briefly, the cultured GCs from D280 hens SYFs were treated with H_2_O_2_ in a gradient concentrations from 50 to 150 μM to induce oxidative damage. Based on the evaluation of cell proliferation and apoptosis rates, the dose of 100 μM H_2_O_2_ was chosen as the optimal concentration in the subsequent experiments.

#### 2.3.2 Experiment 2: GSPs Dose Screening

Likewise, the cultured GCs from D280 hens SYFs were treated with GSPs in a gradient concentrations from 1 to 100 μM. Based on the evaluation of cell proliferation rates, the dose of 10 μM GSPs was adopted as the optimal concentration for the following experiments.

#### 2.3.3 Experiment 3: Treatment With Different Activators or Inhibitors

After pretreatment with GSPs (10 μM) for 24 h, GCs were washed in M199 and incubated with a medium containing 100 μM H_2_O_2_ for different time. GCs were treated with DC661 (a lysosomal protease inhibitor, 5 μM), LY294002 (LY, a broad-spectrum inhibitor of PI3K, 20 μM), AT7867 (an AKT inhibitor, 10 μM), Sirtinol (an sirtuin (SIRT) inhibitor, 100 μM), SRT1720 (an SIRT activator, 100 μM), 3-MA (an autophagy inhibitor, 10 mM), Z-VAD-FMK (ZVF, a pan-caspase inhibitor, 50 μM) or (−)-DHMEQ (an antioxidant inhibitor, 10 μg/ml) for 1 h before H_2_O_2_ exposure.

### 2.4 Morphological Observation

GCs were fixed in 4% neutral paraformaldehyde solution for 2 h at 4°C. After fixation, tissues and cells were rinsed with running water, and the cells were used for subsequent immunohistochemistry. The tissues were dehydrated by graded ethanol and immersed in 60°C paraffin for more than 4 h and embedded. The paraffin section was prepared at 4 μm thickness for immunohistochemistry (IHC), bromodeoxyuridine (BrdU, Sigma-Aldrich, WI, United States) incorporation and TUNEL assay. Hematoxylin and eosin (H&E) staining was carried out according to a conventional protocol. Immunofluorescence (IF) staining was referred to a previous method (Zhou et al., 2021). The primary antibody used for the IF was rabbit anti-LC3B (1:200, ET1612-91, HuaAn Biotechnology Co., Hangzhou, China). For detection of the proliferating cells, EdU (20 μM 5-ethynyl-2′ -deoxyuridine, C0071S, Beyotime Biotechnology) was added into the culture GCs for 2 h. Cells were counterstained by 4′,6-diamidino-2-phenylindole (DAPI) for 5 min. Mounted slides were captured using an Olympus IX70 microscope.

TUNEL staining was performed in the cultured cells via a BrightGreen Apoptosis Detection Kit (A112-03, Vazyme Biotech, Nanjing, China) according to the manufacturer’s instructions. Mounted slides were captured using an Olympus IX81 microscope.

For BrdU incorporation assay, SWFs were incubated for 24 h with 10 μg/ml BrdU. After 72 h of treatment, SWFs were collected for subsequent determinations. IF was performed as previously reported (Zhou et al., 2021). The fluorescence images of the slides were visualized using a fluorescence microscope (Olympus IX70, Tokyo, Japan).

### 2.5 Cell Viability Assay

Cell Counting Kit-8 (CCK-8; Fudebio, Hangzhou, China, FD3788) was used to measure cell viability. GCs were seeded in 96-well plates and grew to 90% confluency for 2 days. CCK-8 assay reagent (10 μL) was added to each well containing 200 μL medium after different treatments. Then, GCs were incubated in the dark for 4 h at 38.5°C. The formation of formazan was assessed by optical density at 450 nm under a microplate spectrophotometer.

### 2.6 Detection of ROS

ROS level was detected by ROS Assay Kit (S0033M, Beyotime Institute of Biotechnology, Hangzhou, China) according to the manufacturer’s instructions. Green fluorescence was emitted upon excitation at 488 nm. GCs were captured with an Olympus microscope (IX70). The results were calculated as fluorescence intensity in each GC by using the ImageJ software (National Institutes of Health, Bethesda, MD, United States).

### 2.7 Determination of Total Antioxidation Capability

Total Antioxidant Capability Assay Kit (Nanjing Jiancheng Bioengineering Institute, Nanjing, China) was used to determine the total antioxidation capability (T-AOC) according to the manufacturer’s instruction. The cells were digested with EDTA-trypsin for 2 min and terminated using the DMEM containing 5% FCS. Briefly, cellular homogenates were centrifuged at 12,000 g at 4°C for 10 min, and protein concentration was determined using a BCA Protein Assay Kit (Nanjing Jiancheng Bioengineering Institute, Nanjing, China) according to the instruction. The reduction of Fe^3+^-TPTZ was then detected at λ593 nm.

### 2.8 Acridine Orange Staining

GCs were stained with 1 μg/ml acridine orange for 15 min at 37°C. In acridine orange-stained cells, the cytoplasm and nucleus emit green fluorescence, whereas the acidic compartments shine bright red (Ming et al., 2018). The green (510–530 nm) and red (650 nm) fluorescence emission illuminated with blue (488 nm) excitation light were visualized under an Olympus IX70 microscope.

### 2.9 RNA Extraction and RT-qPCR

Total RNA was extracted from GCs with Trizol reagent (Invitrogen Co., Carlsbad, CA, United States) according to the manufacturer’s instruction. The cDNA was synthesized using the RevertAid First Strand cDNA Synthesis Kit (Thermo Fisher Scientific, San Jose, CA, United States) following the manufacturer’s instruction. The reverse transcription product was diluted at 1:10 and then used as a cDNA template for RT-qPCR analysis. Relative expression of the target genes was determined by RT-qPCR that was carried out on ABI 7500 HT Real-Time PCR machine (Applied Biosystems, Foster City, CA, United States) in a 10 µL volume using AceQ Universal SYBR qPCR Master Mix (Vazyme., Nanjing, China). Sequences of the primers were provided in Table 1. All samples were normalized with the average of β-actin and *GAPDH* using the comparative cycle threshold method [2^−(△) (△) Ct^).

**TABLE 1:** Primers for PCR analysis.

Genes	Accession no.	Primer sequence (5′–3′)
*Bnip3*	XM_421829.6	TCA​GCC​CGC​AGG​AGG​AGA​AC
		CCA​CGC​TGT​TTC​CAT​TGC​CAT​TC
*Atg12*	XM_004949628.3	TGC​CAG​GTG​ACA​GTC​TCA​GTC​C
		AGT​GCC​ACT​TAC​AGG​AGA​CAG​AGG
*Map1lc3b*	NM_001031461.1	CTG​GTG​AAC​GGA​CAC​AGC​ATG​G
		AAG​CCG​TCC​TCG​TCC​TTC​TCG
*Atg7*	NM_001030592.1	AGG​CTC​GGA​AGG​ATG​TGG​CTA​C
		CCA​GGG​CAG​CAT​TGA​TGA​CCA​G
*Pik3c3*	XM_004949052.3	ACT​CAG​CAG​AGG​GAC​CCA​AAG​AC
		TGA​ACC​AGC​CGA​TCC​ACA​AAT​GTC
*RAB7*	NM_001389626.1	GAC​AAG​AAC​GAC​CGA​GTG​AAG​GC
		AAG​AGG​GGC​TGT​GTG​TGT​TTG​AAG
*Becn1*	XM_015299595.2	GGT​TCA​TCC​CAC​CAG​CCA​GAA​TG
		TGC​CTC​CAT​CTG​ATG​CCT​CTC​C
β-actin	NM_205518	ACA​CCC​ACA​CCC​CTG​TGA​TGA​A
		TGC​TGC​TGA​CAC​CTT​CAC​CAT​TC
*GAPDH*	NM_204305.1	TCA​CAG​CCA​CAC​AGA​AGA​CG
		ACT​TTC​CCC​ACA​GCC​TTA​GC

### 2.10 Western Blot Analysis

#### 2.10.1 Total Protein Extraction

The SWFs from D580 and D280 hens were homogenized using 500 μL ice-cold RIPA (P1003B, Beyotime, Jiangsu, China) supplemented with 1 mM phenylmethanesulfonyl fluoride (Beyotime, Shanghai, China). The cultured GCs were digested with EDTA-trypsin for 2 min and homogenized by 50 μL RIPA containing 1 mM phenylmethanesulfonyl fluoride.

#### 2.10.2 Nuclear Protein and Cytoplasmic Protein Extraction

The cultured GCs were digested with EDTA-trypsin for 2 min. The nuclear protein and cytoplasmic protein extraction kit was used to extract nuclear protein and cytoplasmic protein (P0027, Beyotime Institute of Biotechnology, Hangzhou, China) according to the manufacturer’s instructions.

#### 2.10.3 Protein Quantification

The total protein was quantified by BCA Protein Assay Kit (Nanjing Jiancheng Bioengineering Institute, Nanjing, China). Samples of 22 μg of protein were applied to a 10% SDS–polyacrylamide gel electrophoresis and the proteins were transferred onto a polyvinylidene difluoride (PVDF) membrane (0.22 μm, Millipore, Bedford, United States) after running for 45 min at 120 V. After blocking with 5% skim milk, the PVDF membrane was incubated with corresponding primary antibodies including rabbit anti-LC3B (1:500, ET1701-65), anti-SQSTM1 (1:500, R1309-8), anti-Bcl2A1 (1:500, ET1610-20), anti-caspase 3 (1:500, ER 1802-42), anti-PCNA (1:500, R1306-5), anti-AKT (1:500, EM40507), anti-p-AKT (1:500, ET1607-73), anti-FoxO1 (1:500, ET1608-25, HUABIO, Hangzhou, China) and anti-ac-FoxO1 (1:500, A17406, ABclonal, Wuhan, China). Next, the membrane was incubated with the secondary antibodies. Blots were washed three times and visualized using FDbio-Femto ECL Substrate Kit (FD8030, FDbio, Hangzhou, China). For protein quantification, Gel-Pro Analyzer (Media Cybernetics, United States) was used to quantify and analyze images with β-actin as the internal control.

### 2.11 Transmission Electron Microscopy

GCs were fixed in 2.5% glutaraldehyde for 24 h at 4°C and dehydrated in ethyl alcohol and acetone. Then GCs were embedded in LX 112 epoxy resin. Sections of 70-90 nm thickness were cut with an ultramicrotome (Leica Microsystems GmbH, Wetzlar, Germany) and mounted on formvar-coated copper grids. The samples were observed and photographed by a Tecnai G2 Spirit (FEI Company, Hillsboro, United States) at various magnifications after staining.

### 2.12 Statistical Analysis

All experiments were repeated three times. Data were expressed as the mean ± standard error of the means and analyzed with post hoc Dunnett’s test and independent samples t-test or by One-way ANOVA and Two-way ANOVA via the GraphPad Prism8 software. *p* < 0.05 was statistically significant.

The experimental design was provided in Supplementary Figure S1.

## 3 Results

### 3.1 Comparison of Autophagy in Ovaries Between D280 and D580 Hens

**FIGURE 1:**
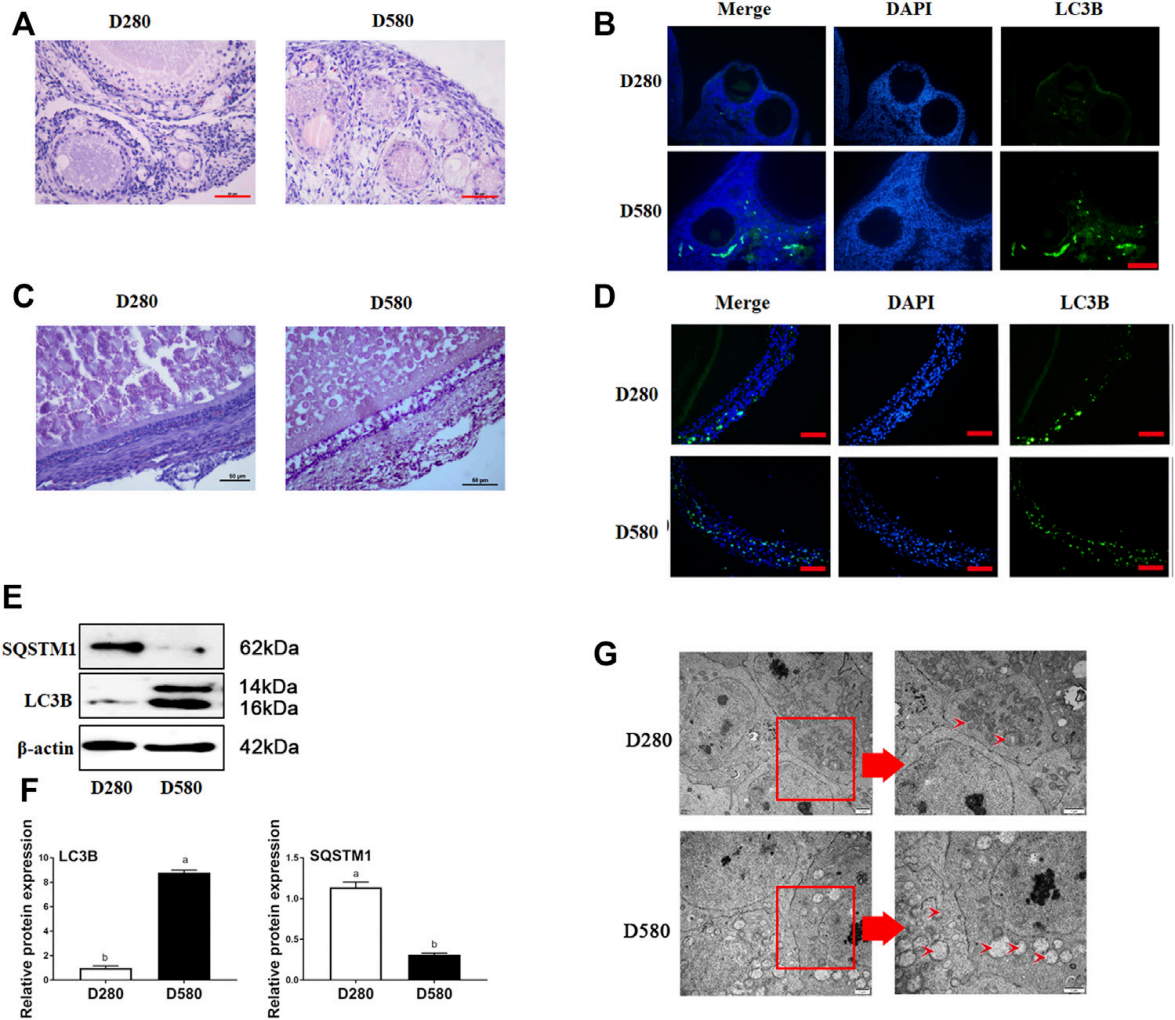
The morphological changes of ovarian and follicle tissues in different ages hens. **(A)** Representative morphology of ovarian tissues in hens D280 and D580. Scale bar: 50 µm. **(B)** The expression of LC3B in ovarian tissues from hens aged D280 and D580. Scale bar: 20 µm. **(C)** Representative morphology of SWF in hens D280 and D580. Scale bar: 50 µm. **(D)** The expression of LC3B in SWF from hens aged 280 and 580 days. LC3B protein is mainly expressed in GCs via IF. Scale bar: 20 µm **(E)** The expression of SQSTM1 and LC3B by Western blot. **(F)** Relative expression of proteins related to autophagy in GCs. **(G)** The ultramicrostructure of the follicle in hens aged 280 and 580 days. An enlarged view of parts in the box on right. Scale bar: 1 µm. Arrows: autophagic vesicle.

The results of H&E and immunofluorescence assay showed that the formation of autophagy was remarkably enhanced in ovaries and follicles collected from D580 hens as compared with D280 hens (Figures 1A–D). Meanwhile, the LC3B, positive staining of the autophagy-related biomarkers, was concentrated almost in the granulosa layers (Figures 1B,D). Notably, Western blot analysis of LC3B expression was increased 88.89%. SQSTM1 degradated 75% in aging hens. These results further confirmed that autophagy within ovarian GCs (Figures 1E,F). TEM also showed that more autophagic vacuoles appeared in the aging follicles (Figure 1G).

### 3.2 Reduced Autophagy After GSPs Treatment in D580 Hen SWFs

**FIGURE 2:**
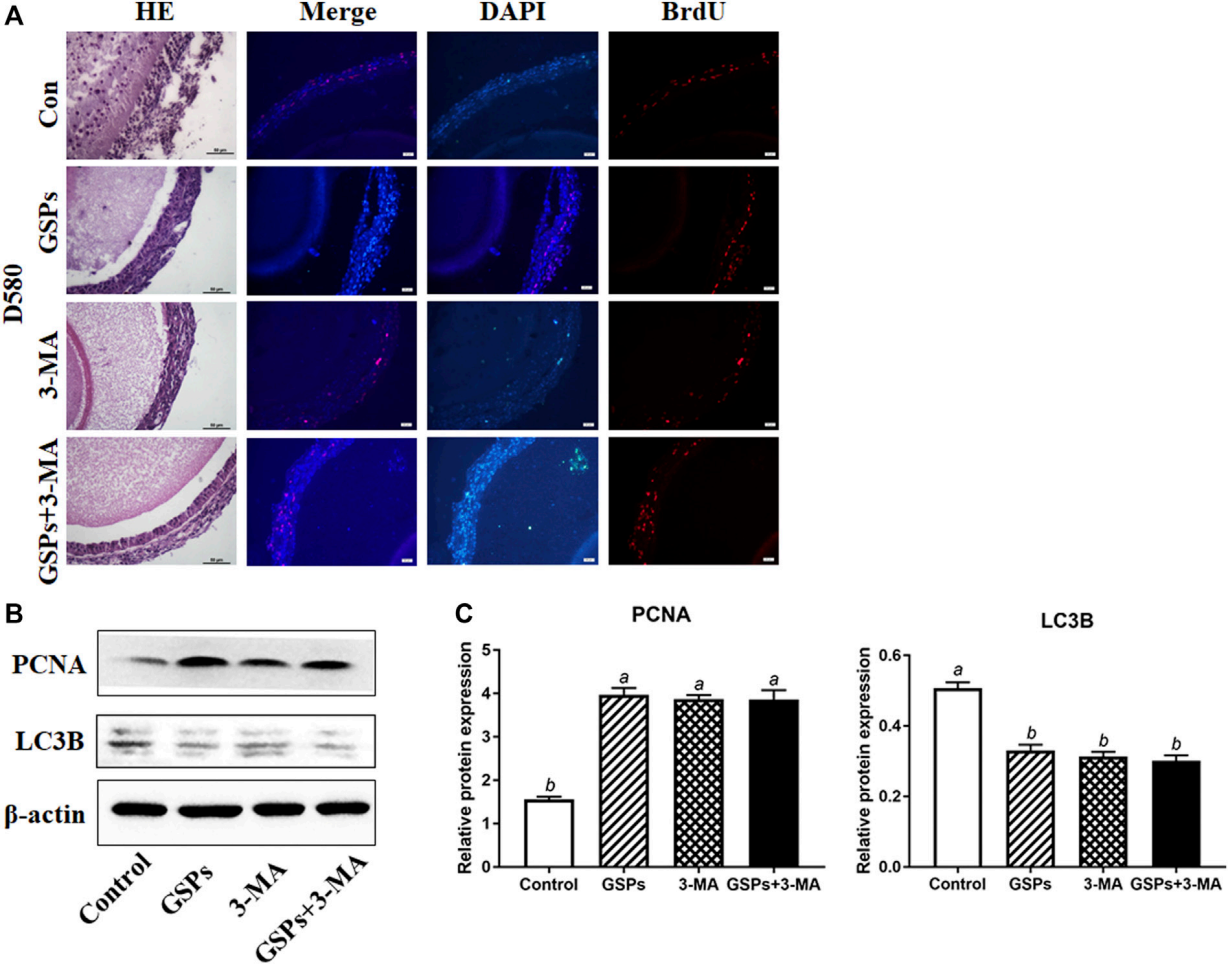
GSPs represses autophagy in follicle from D580 hens. **(A)** Effect of GSPs or 3-MA (an inhibitor of autophagy, 10 mM) on the proliferation of SWFs from D580 hens. Scale bar of HE: 50 µm. Scale bar of IF: 20 µm. **(B)** The immunoblotting detection of PCNA and LC3B in SWFs from D580 chickens were determined by Western blotting. **(C)** The expression of PCNA and LC3B in SWFs from D580 chickens. Bars with different superscripts are statistically different (*p* < 0.05). Values are the means ± SEM (*n* = 3).

The structure of SWFs in D580 hen ovarian tissues displayed a loose and irregular arrangement of the granulosa cells. This structural deterioration was remarkably alleviated by 5 μg/ml (8.5 μM) GSPs treatment. This effect was similar to the treatment of an autophagy inhibitor 3-MA (10 mM). Both GSPs and 3-MA treatment increased GC proliferation in SWFs through BrdU staining assay (Figure 2A). Compared with control group, treatment with GSPs or 3-MA alone significantly increased the expression of PCNA at least 60% and decreased 40% on the expression of LC3B in SWFs from D580 hens (Figures 2B,C).

### 3.3 Reduced Autophagy After GSPs Treatment in D580 Hen GCs

**FIGURE 3:**
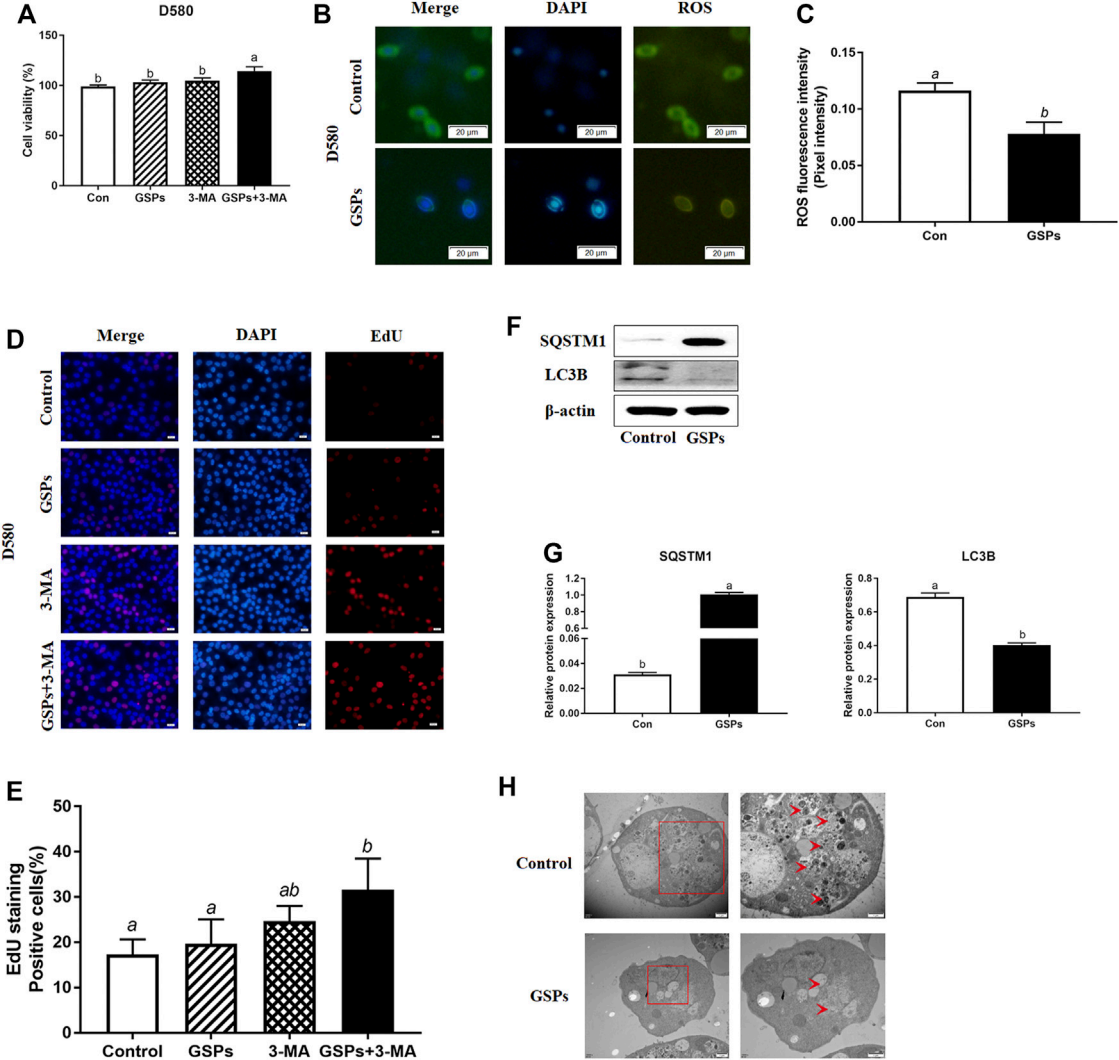
GSPs represses autophagy in cultured GCs from D580 hens. **(A)** Cell viability was examined on GCs, which were treated with GSPs or 3-MA. **(B)** ROS fluorescence staining in GCs from D580 chicken SYFs. Scale bar: 20 µm. **(C)** The fluorescence intensity of ROS in GCs. The optical density was quantified by ImageJ software. Experiments were repeated in three times, and three fields of each coverslip were selected in random for analysis. **(D)** Effect of GSPs or 3-MA on the proliferation of GCs from D580 chicken SYFs by EdU staining. Scale bar: 20 µm. **(E)** The positive cells rate in EdU staining. **(F)** The immunoblotting detection of SQSTM1 and LC3B in GCs from D580 SYFs were determined by Western blotting. **(G)** The expression of SQSTM1 and LC3B in GCs from D580 SYFs. **(H)** The ultrastructure of GCs of the follicle in hens aged 580 days with/without GSPs. An enlarged view of parts in the box on right. Scale bar: 1 µm. Arrows: autophagic vesicle. Scale bar: 1 µm. Bars with different superscripts are statistically different (*p* < 0.05). Values are the means ± SEM (*n* = 3).

GSPs treatment did not enhance the cell viability, but the treatment with both GSPs and 3-MA improved cell viability (Figure 3A). Compared with the control, treatment of GSPs significantly showed lower ROS levels in GCs from D580 hen follicles (Figures 3B,C). By EdU incorporation assay, no difference in cellular proliferation when GSPs or 3-MA was added separately, but there was increased cellular proliferation adding both GSPs and 3-MA (Figures 3D,E). GSPs alleviated autophagy of GCs from D580 hens. GSPs decreased the expression of LC3B protein by 57.14% and increased the expression of SQSTM1 protein over 3 times (Figures 3F,G). As shown in Figure 3H, treatment of GSPs significantly decreased the number of autophagic vesicles in GCs from D580 hen GCs.

### 3.4 Establishment of the Aging Model of GCs and GSPs Dose Screening

**FIGURE 4:**
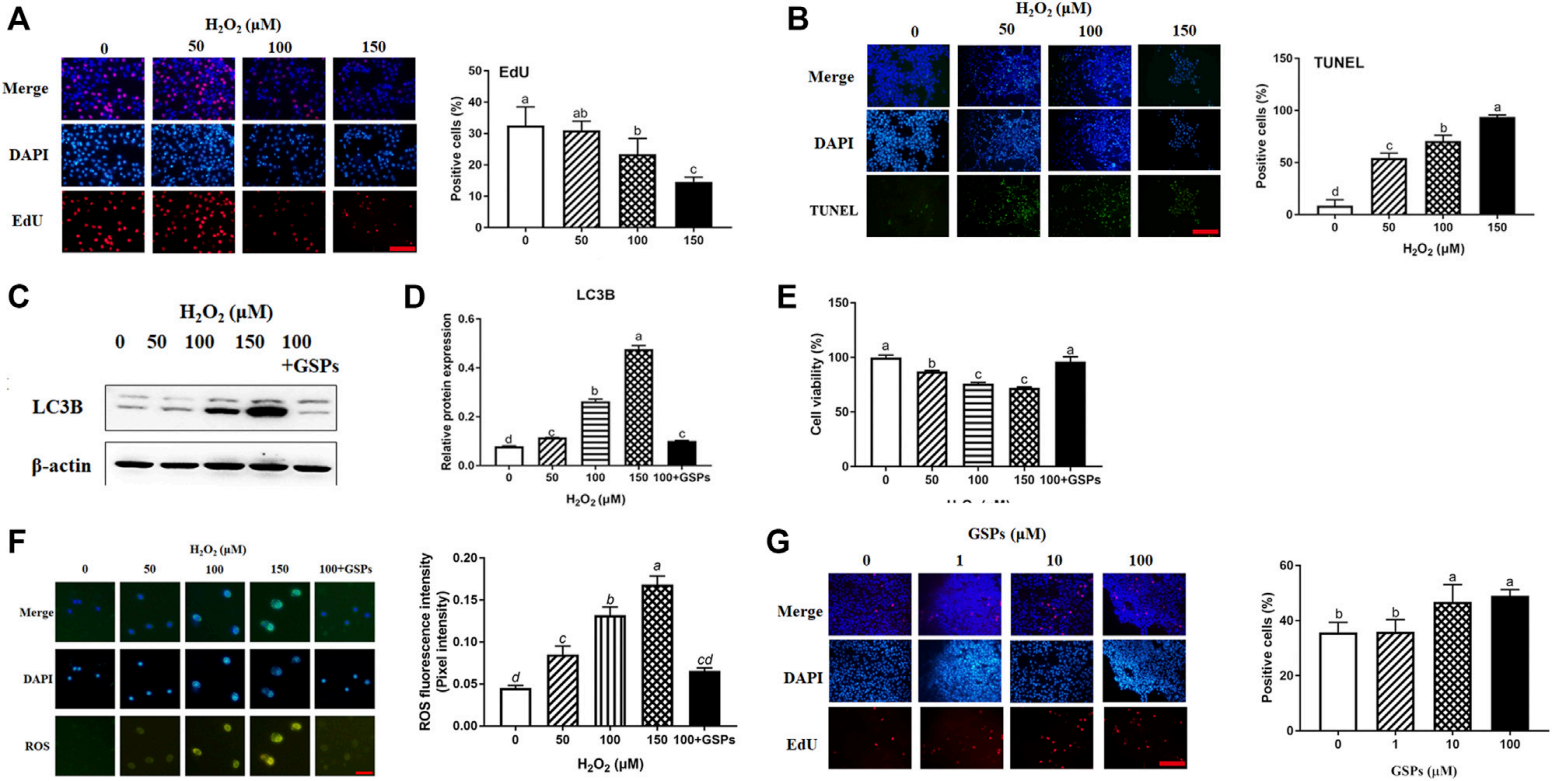
Effects of GSPs or H_2_O_2_ changes of GCs via EdU incorporation and TUNEL staining. **(A)** Effect of H_2_O_2_ on proliferation of granulosa cells. Scale bar: 20 µm. **(B)** Effect of H_2_O_2_ on apoptosis of granulosa cells. Scale bar: 20 µm. **(C,D)** The immunoblotting detection of LC3B in GCs from D280 chicken SYFs. **(E)** Cell viability was examined as described above. **(F)** The fluorescence intensity of ROS in GCs was observed by a fluorescence microscope. Scale bar: 20 µm. **(G)** Effect of GSPs on the proliferation of GCs. Bars with different superscripts are statistically different (*p* < 0.05). Scale bar: 20 µm.

H_2_O_2_ was used to establish aging model of GCs from D280 hen SYFs. The effect of H_2_O_2_ on the proliferation of GCs was detected by EdU incorporation. The result showed that H_2_O_2_ treatment significantly inhibited the proliferation of GCs. Treatment with 100 μM H_2_O_2_ markedly reduced the proliferation of GCs by 28.22% and higher H_2_O_2_ (150 μM) further decreased cell proliferation by 55.21% (Figure 4A). Compared with the control, treatment with 100 μM H_2_O_2_ significantly enhanced GCs apoptosis rate. However, the apoptosis rate was more than 90% after the treatment of 150 μM H_2_O_2_ (Figure 4B). Western blot experiment showed that the LC3B expression displayed a dose-dependent manner in the cultured GCs after H_2_O_2_ treatment and was significantly inhibited by GSPs treatment (Figures 4C,D). The cell viability displayed a dose-dependent manner decrease in the cultured GCs after H_2_O_2_ treatment and this downward trend was alleviated by GSPs (Figure 4E). The ROS fluorescence intensity displayed a dose-dependent manner increase in the cultured GCs after H_2_O_2_ treatment. GSPs effectively alleviated the accumulation of ROS caused by the 100 μM H_2_O_2_ (Figure 4F). Thus 100 μM H_2_O_2_ was chosen to establish aging model of GCs. Then GSPs dose was screened. Treatment with 10 or 100 μM GSPs remarkably increased the positive cells (Figure 4G). The 10 μM GSPs was chosen for the following experiments.

### 3.5 Effect of GSPs on GCs From Oxidative Damage via Inhibiting Autophagy

**FIGURE 5:**
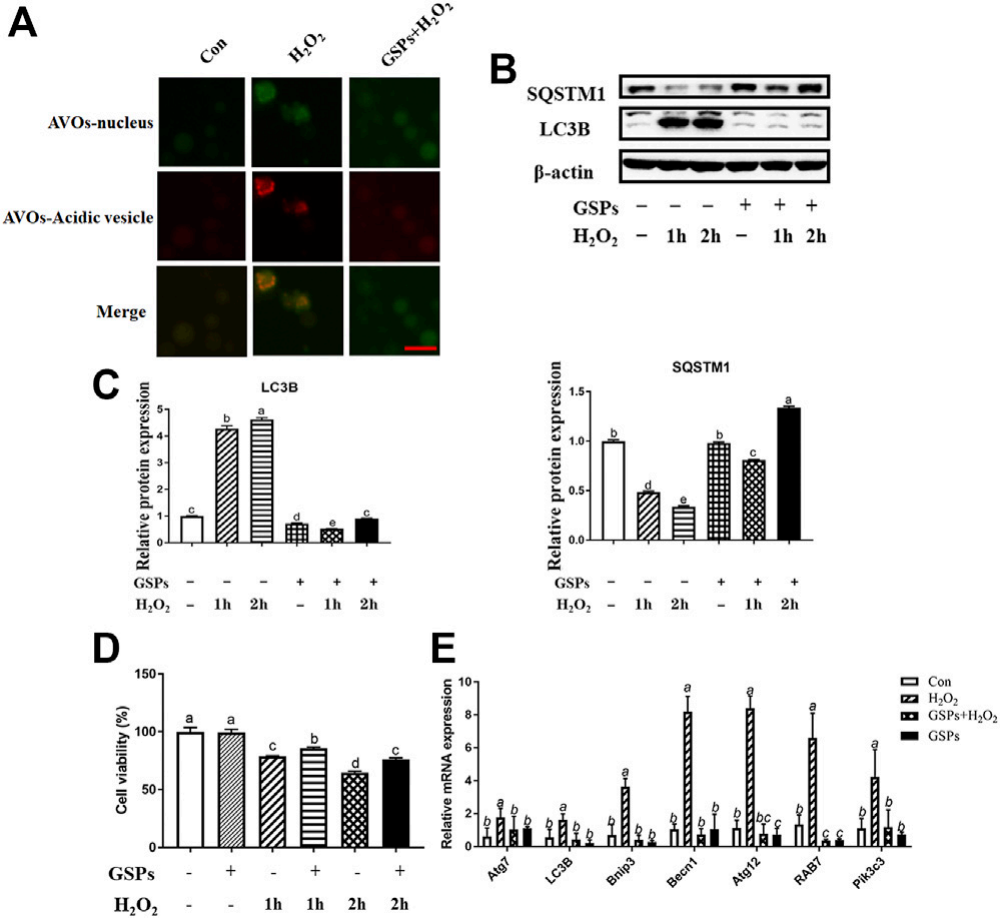
The effect of GSPs on H_2_O_2_ -induced autophagy in cultured GCs. **(A)** The acridine orange staining was used to detect the AVOs (acidic vesicular organelles, red) in H_2_O_2_-treated 2 h GCs from D280 SYFs to show a morphological characteristic of autophagy. Scale bar: 20 µm. **(B)** The expression of LC3B and SQSTM1 in GCs was determined by Western blotting. **(C)** The LC3B and SQSTM1 were quantified by densitometric analysis. **(D)** Cell viability using the CCK-8 assay. **(E)** The mRNA levels of autophagy-related genes in GCs were measured by RT-qPCR. Bars with different superscripts are statistically different (*p* < 0.05). Values are the means ± SEM (*n* = 3).

**FIGURE 6:**
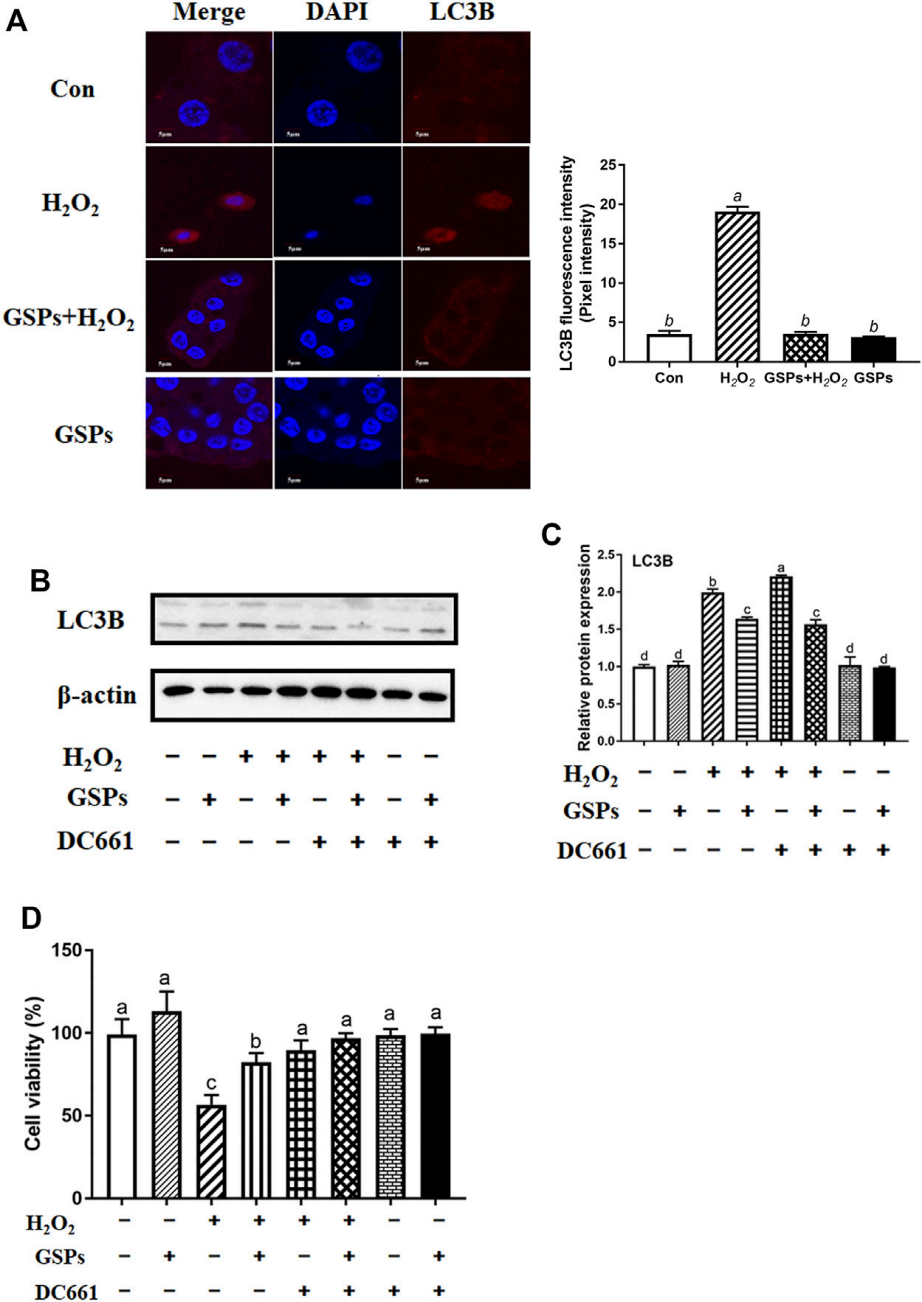
Effects of GSPs and DC661 on H_2_O_2_-induced decline of autophagy. **(A)** LC3B-positive cells were compared in the control, H_2_O_2_, GSPs, and H_2_O_2_+GSPs groups. Scale bar: 5 µm. **(B,C)** Relative expression of proteins related to autophagy. DC661 (lysosomal protease inhibitor, 5 μM). **(D)** Cell viability using the CCK-8 assay. Bars with different superscripts are statistically different (*p* < 0.05). Values are the means ± SEM (*n* = 3).

As shown in Figure 5A, GCs pretreated with 10 μM GSPs for 24 h showed a marked reduction in the number of AVOs after being treated with 100 μM H_2_O_2_. Correspondingly, the Western blot experiment showed that the LC3B expression and SQSTM1 degradation after H_2_O_2_ exposure were significantly inhibited by GSPs treatment (Figures 5B,C). Statistic results showed that the LC3B expression was increased over 80.95% in H_2_O_2_-teated 1 h or 2 h. Moreover, the decreased viability of GCs caused by H_2_O_2_ was significantly increased in the presence of GSPs (Figure 5D). GSPs alleviated the effect of 100 μM H_2_O_2_-teated 2 h on GCs better than H_2_O_2_-teated 1 h. The results of the RT-qPCR analysis showed that pretreatment of GSPs reduced the expression of *Rab7, Pik3c3, Map1lc3b, Atg7, Becn1, Bnip3,* and *Atg12* in the H_2_O_2_-treated for 2 h in the GCs compared with the H_2_O_2_ group (model control). However, GSPs alone did not decrease the expression of autophagy-related genes (Figure 5E). IF showed that LC3B was increased in H_2_O_2_-treated GCs (Figure 6A). However, DC611 alone did not increase the accumulation of LC3B and cell viability (Figures 6B–D).

### 3.6 Effect of GSPs on GCs From Oxidative Damage via Repressing Autophagy

**FIGURE 7:**
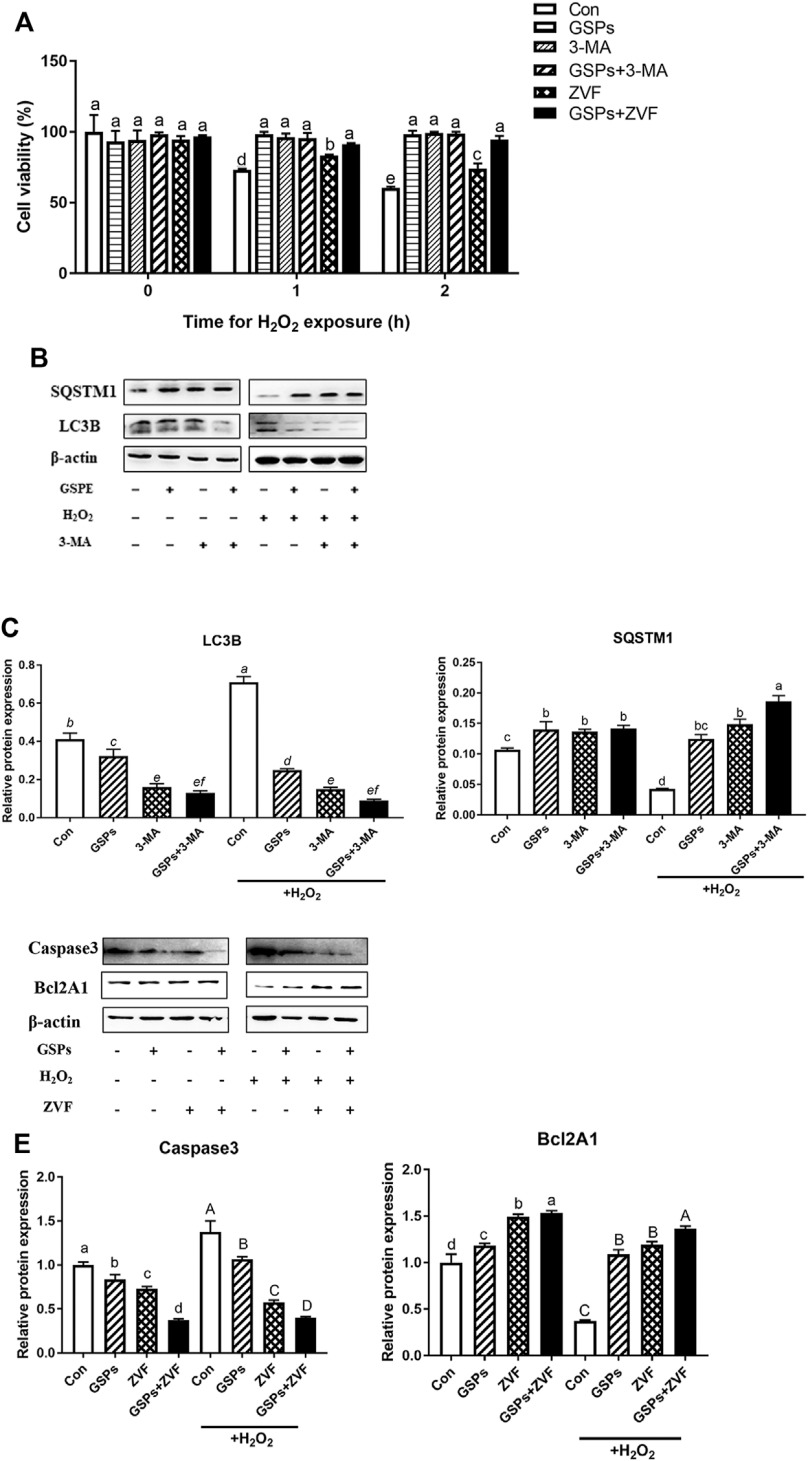
GSPs prevent oxidative stress-induced GC injury *via* preferentially inhibiting autophagic death. **(A)** Cell viability was determined using the CCK-8 assay. Z-VAD-FMK (ZVF, a well-known pan-caspase inhibitor, 50 μM). **(B)** GCs pretreated with 10 μM GSPs for 24 h were then rinsed in PBS and exposed to 100 μM H_2_O_2_ for 2 h. To block the autophagic flux, 3-MA (10 mM) was added before H_2_O_2_ exposure. Western blotting showed expression levels of LC3B and SQSTM1. **(C)** Quantification of immunoblot signals for SQSTM1 and LC3B accumulation. **(D)** GCs pretreated with 10 μM GSPs for 24 h were then rinsed in PBS and exposed to 100 μM H_2_O_2_ for 2 h. To block the apoptosis, ZVF (50 μM) was added before H2O2 exposure. **(E)** The protein levels of Caspase 3 and Bcl2A1 were evaluated. Bars with different superscripts are statistically different (*p* < 0.05). Values are the means ± SEM (*n* = 3).

Both GSPs and 3-MA treatment restored the viability of GCs after H_2_O_2_ treatment for 2 h H_2_O_2_ treatment also caused cell apoptosis. However, treatment of ZVF did not completely alleviate the viability of GCs (Figure 7A). Western blot experiment showed that treatment of GSPs or 3-MA reduced the expression of LC3B over 70% and enhanced the expression of SQSTM1 over 110% in the cultured GCs with H_2_O_2_ treatment for 2 h (Figures 7B,C). Moreover, pretreatment of GSPs or ZVF reduced the expression of Caspase 3 and enhanced the expression of Bcl2A1 in the cultured GCs with or without H_2_O_2_ treatment for 2 h (Figures 7D,E).

### 3.7 Changes in ROS Scavenging After GSPs Treatment

**FIGURE 8:**
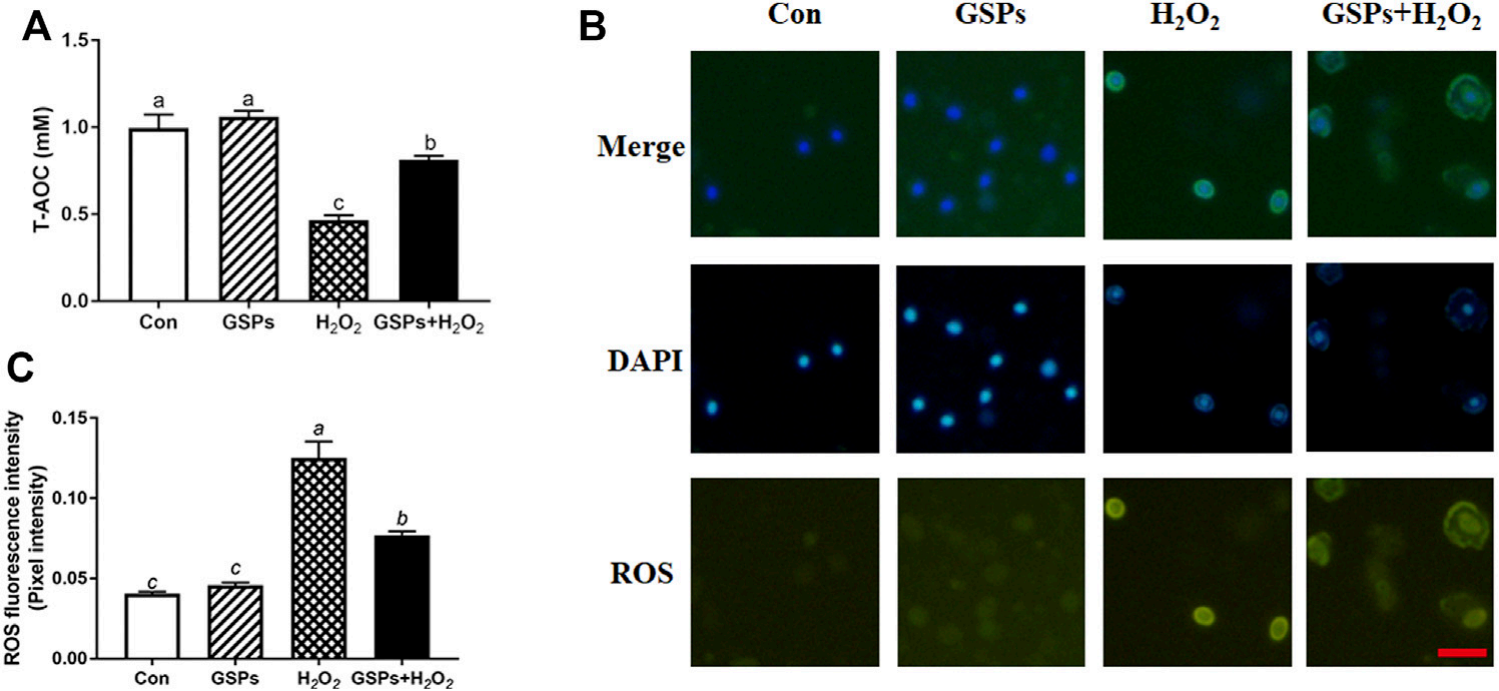
GSPs reduce the generation of ROS in GCs through oxidative stimulation. **(A)** GCs pretreated with or without 10 μM GSPs for 24 h and cultured in H_2_O_2_ (100 μM) 2 h later, T-AOC was detected. **(B)** The fluorescence intensity of ROS in GCs. Scale bar: 20 µm. **(C)** Quantification of intracellular ROS levels. The optical density was calculated in each GC with ImageJ software. Experiments were repeated in triplicate, and three fields of each coverslip were selected in random for counting. Bars with different superscripts are statistically different (*p* < 0.05). Values are the means ± SEM (*n* = 3).

**FIGURE 9:**
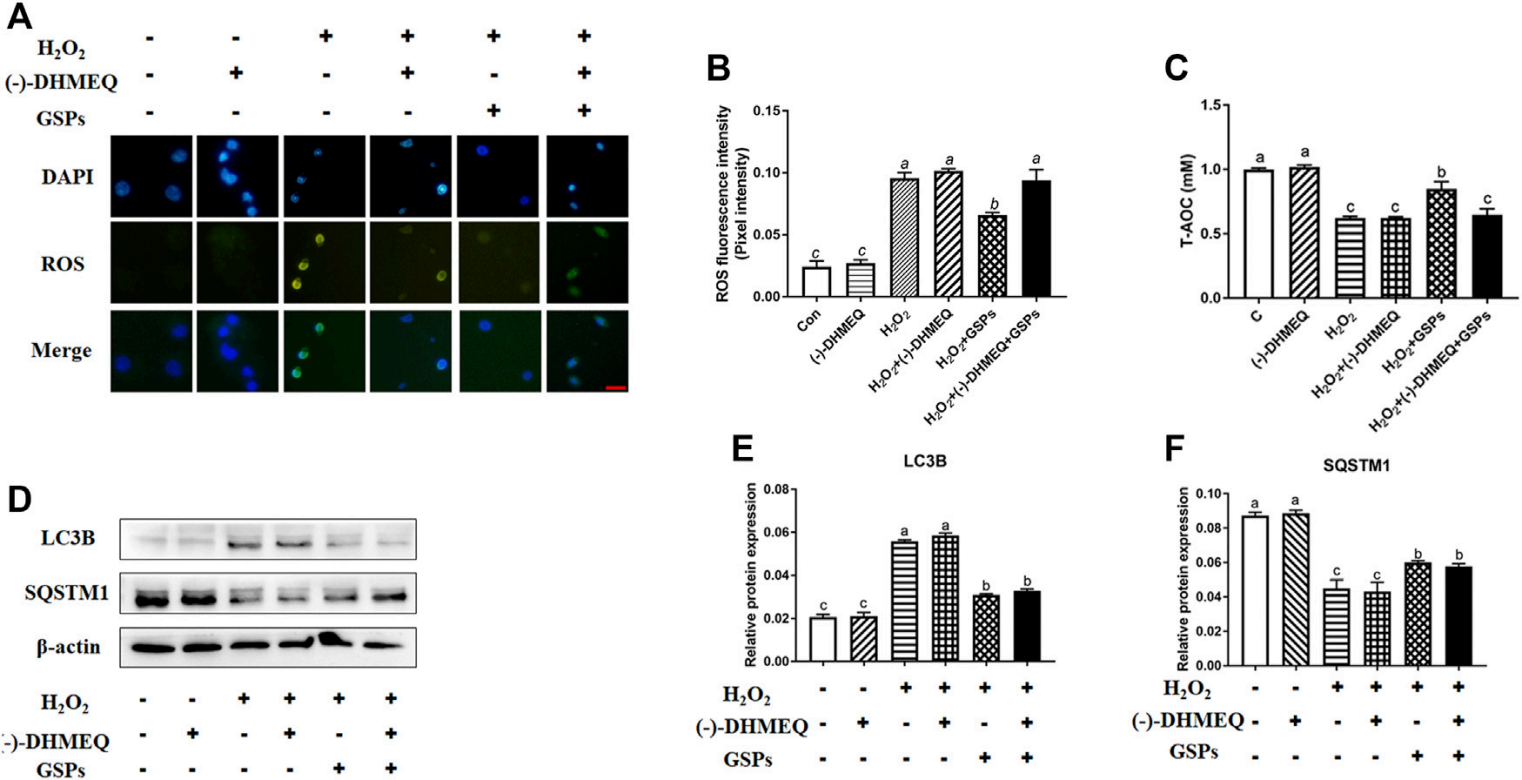
The inhibitory effect of GSPs on H_2_O_2_-induced autophagic GC death is not entirely dependent on the scavenging of reactive oxygen species. **(A)** GCs were exposed to GSPs and/or (−)-DHMEQ (an antioxidant inhibitor, 10 μg/ml) as described above. The level of ROS was detected by dichlorofluorescein fluorescence (green), and the nuclei were stained with DAPI (blue). Scale bar: 20 µm. **(B)** ImageJ software was used to quantify the optical density of ROS. **(C)** T-AOC was measured with GSPs and/or (−)- DHMEQ. **(D)** GCs were exposed to GSPs and/or (−)-DHMEQ (an antioxidant inhibitor, 10 μg/ml) as described above. The expression of LC3B and SQSTM1 in GCs were determined by Western blotting. **(E,F)** The LC3B and SQSTM1 were quantified by densitometric analysis. Bars with different superscripts are statistically different (*p* < 0.05). Values are the means ± SEM (*n* = 3).

The GSPs improved the T-AOC of GCs upon H_2_O_2_ exposure (Figure 8A). ROS staining showed that GSPs reduced ROS concentration. However, GSPs did not completely obliterate intracellular ROS (Figures 8B,C). Different in the treatment of (-)-DHMEQ, GSPs decreased ROS concentration in H_2_O_2_-treated GCs (Figures 9A,B). Meanwhile, GSPs also alleviated the decrease of T-AOC caused by H_2_O_2_, while (−)-DHMEQ has no such effect in GCs from D280 chicken SYFs (Figure 9C). GSPs increased the expression of SQSTM1 in H_2_O_2_-treated GCs. Meanwhile, GSPs decreased the expression of LC3B in H_2_O_2_-treated GCs (Figures 9D–F).

### 3.8 Role of AKT Pathway in GSPs-Induced Change in Autophagy

**FIGURE 10:**
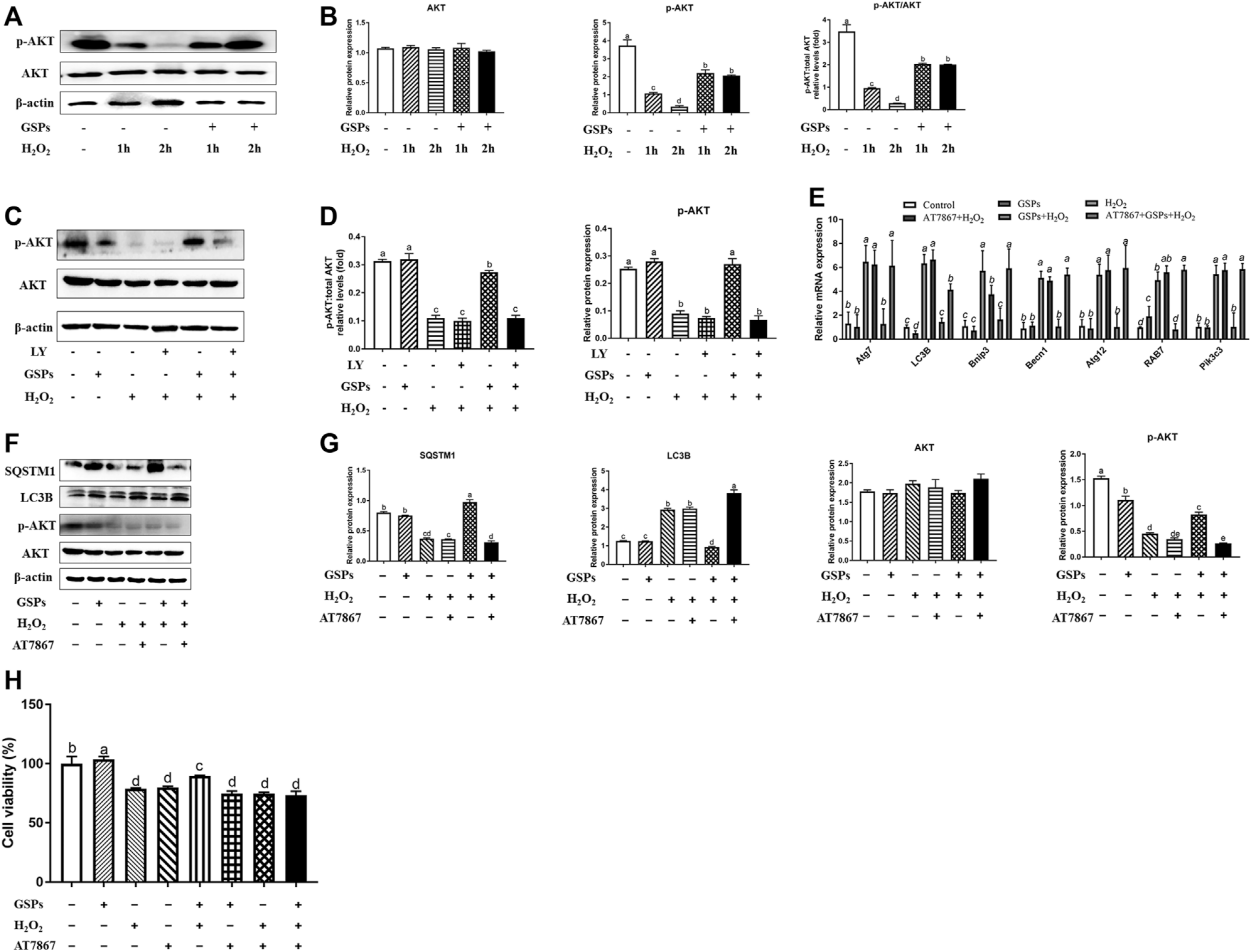
GSPs counteract H_2_O_2_-induced GC autophagy via the PI3K-AKT pathway. **(A,B)** GCs subjected to H_2_O_2_ (100 μM) incubation for 1 or 2 h after culturing with 10 μM GSPs for 24 h. Western blotting was used to determine the expression of phosphorylated AKT (p-AKT) and total AKT. **(C,D)** After culturing with 10 μM GSPs with/without LY294002 (LY, a broad-spectrum inhibitor of PI3K, 20 μM) for 24 h. Phosphorylated AKT (p-AKT) and total AKT expression were determined by Western blotting. **(E)** RT-qPCR was performed to measure the mRNA levels of autophagy-related (Atg) genes in GCs. **(F,G)** After culturing with 10 μM GSPs with/without AT7867 (AKT inhibitor, 10 μM) for 24 h. The expression of phosphorylated AKT (p-AKT), total AKT, LC3B, and SQSTM1 were determined by Western blotting. **(H)** CCK-8 assay was used to determine cell viability in GCs. Bars with different superscripts are statistically different (*p* < 0.05). Values are the means ± SEM (*n* = 3).

Western blot analysis showed that 100 μM H_2_O_2_-treated 2 h reduced the p-AKT instead of total AKT protein, while GSPs alleviated this downward trend (Figures 10A,B). Treatment of LY to inhibit PI3K led to the abolishment of GSPs-induced AKT activation (Figures 10C,D). AKT inhibitor AT7867 up-regulated the expression of *Rab7, Pik3c3, Map1lc3b, Atg7, Becn1, Bnip3,* and *Atg12* mRNAs (Figure 10E). AT7867 treatment also reduced the protein expression of p-AKT that was associated with LC3B accumulation and SQSTM1 degradation (Figures 10F,G). Treatment with GSPs alone intensively induced cell viability, in a manner opposite from AT7867, an AKT inhibitor. In addition, the decline in the cell viability by 100 μM H_2_O_2_ treatment were recovered by administration of GSPs. After inhibition of AKT signaling pathway by AT7867, GSPs didn’t restore the decreased cell viability (Figure 10H).

### 3.9 Reduced Autophagy After GSPs Treatment in GCs via SIRT1-FoxO1 Inhibition

**FIGURE 11:**
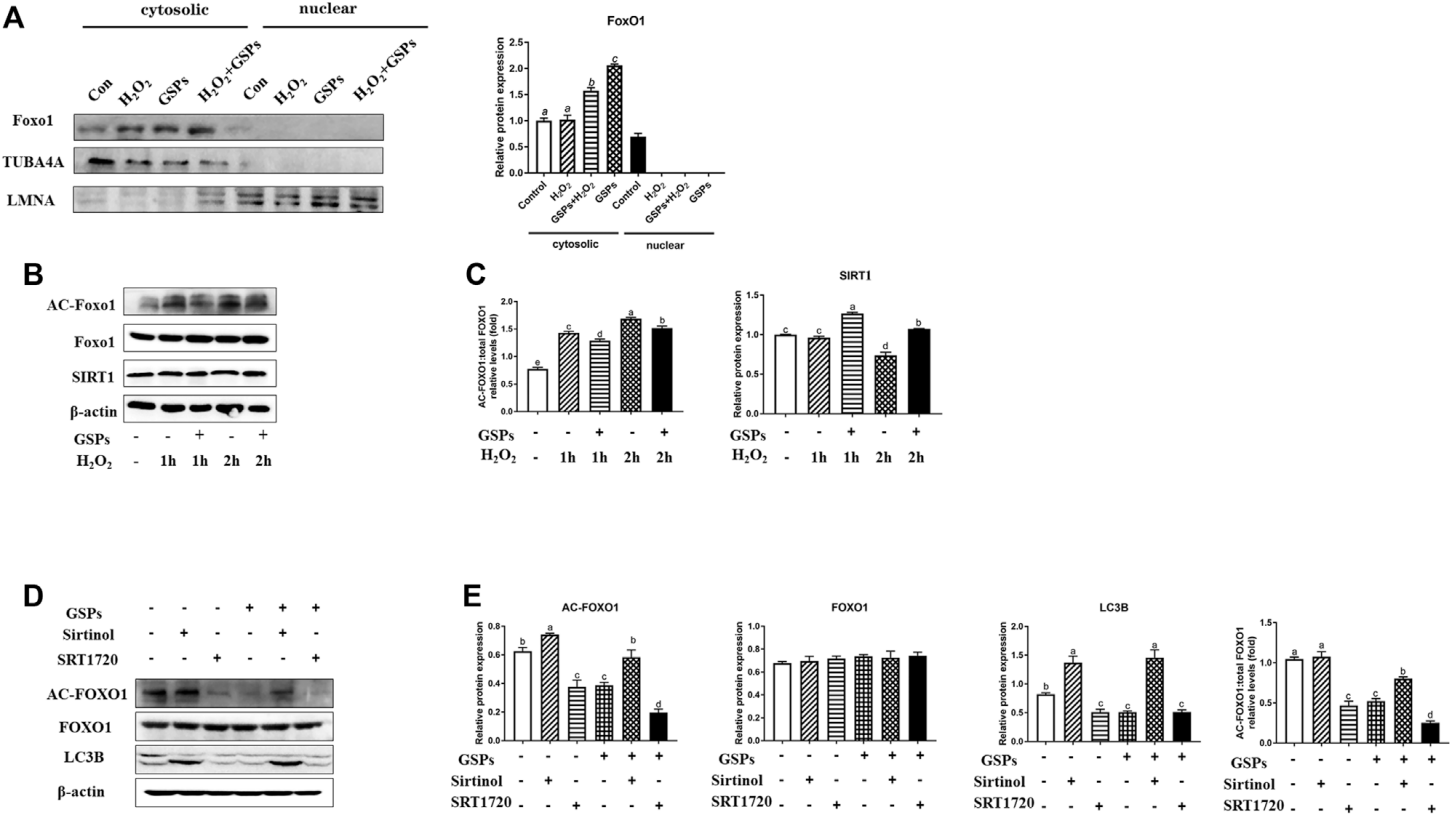
FoxO1 inhibits FoxO1 dependent autophagy in GCs by deacetylation of the GSPs-SIRT1 pathway. **(A)** GCs were exposed to GSPs and/or H_2_O_2_ as described above. The level of FOXO1 in cytosol and nuclei were detected by Western blotting. **(B,C)** GCs subjected to H_2_O_2_ (100 μM) incubation for 1 or 2 h after culturing with 10 μM GSPs for 24 h. The level of acetylated FOXO1 (Ac-FOXO1), total FOXO1, and SIRT1 protein were detected by Western blotting. **(D,E)** The expression of Ac-FOXO1, total FOXO1, and LC3B were quantified by densitometric analysis. Sirtinol (a sirtuin (SIRT) inhibitor, 100 μM), SRT1720 (a SIRT activator, 100 μM). Bars with different superscripts are statistically different (*p* < 0.05). Values are the means ± SEM (*n* = 3).

As shown in Figure 11A, FoxO1 was mainly expressed in the cytoplasm. The expression of AC-FoxO1 was increased in the H_2_O_2_-treated GCs, while treatment of GSPs reduced the expression of AC-FoxO1 and enhanced the expression of SIRT1. The expression of SIRT1 was decreased in the H_2_O_2_-treated GCs for 2 h, while treatment of GSPs induced the expression of SIRT1 (Figures 11B,C). Treatment of GSPs alleviated the increase of AC-FoxO1 protein expression that was induced by H_2_O_2_. Sirtinol (an inhibitor of SIRT1) increased the protein expression of AC-FoxO1 and LC3B. Meanwhile, after treatment by SRT1720 (an activator of SIRT1), the protein expression of AC-FoxO1 and LC3B was decreased in the cultured GCs (Figures 11D,E).

## 4 Discussion

Traditionally, ovary aging in chickens has been simply considered a result of oxidative stress (Tatone et al., 2008; Liu X. T. et al., 2018). The gradual increase of ROS level and the decrease of antioxidant substance level in cells are some of the main reasons for ovarian aging, which in turn triggers follicular atresia and related anovulatory disorders (Agarwal et al., 2012). When the accumulation level of ROS generated by cell metabolism exceeds the scavenging capacity of the cell’s antioxidant system, the original redox balance of the cell is broken, causing extensive oxidative stress and ultimately leading to cell apoptosis (Jinhwan et al., 2015; Orrenius et al., 2017; Liu X. et al., 2018). However, autophagy also occurs in the atretic follicles (Choi et al., 2011). Autophagy signals can be detected in the granulosa layer of the follicle (Choi et al., 2010). More and more evidence suggest that autophagy may serve as a death-promoting pathway and aggravate the damage of GCs under oxidative stress (Nicole et al., 2006; Serke et al., 2009). The mechanisms of autophagy in chicken ovary aging remain poorly understood. In this study, our data showed that GSPs significantly inhibited autophagy and oxidative damage of the follicular GCs by PI3K-AKT/SIRT1-FOXO1 signaling pathway. We proposed a novel role of GSPs in protecting ovarian GCs survival from oxidative damage by inhibition of autophagy.

Using H&E staining, we observed the structure of the D580 chicken ovarian tissues was damaged and the SWFs displayed a loose and irregular arrangement of the granulosa cells, which was consistent with the previous studies (Liu X. T. et al., 2018; Yao et al., 2020). The TEM observation verified that the accumulation of autophagic vesicle in the cytoplasm and such accumulation appeared in D580 chicken SWFs. In brief, the autophagy level did ascend significantly with the aging process. On the other hand, regression of follicles embodies a gradual decrease of proliferation capacity and cell viability. The function of GCs in follicles is more active. GCs degenerate first in the process of aging, performing a decreased activity, decreased proliferation, and increased apoptosis (Liu X. T. et al., 2018; Lin Y. et al., 2018). Our studies revealed that the level of LC3B protein increased sharply and the level of SQSTM1 protein decreased at D580 chicken GCs. In summary, the aging process is accompanied by a significant increase in autophagy.

Supplementation of antioxidants is an effective way to alleviate ovarian oxidative stress thereby relieving autophagy. GSPs is a kind of polyphenol compound, which has a strong anti-oxidation and free radical scavenging effect. At present, the research on the defense mechanism of GSPs is mainly limited to its regulation of apoptosis (Liu X. T. et al., 2018), which has been the main cause of GC death and follicular atresia (Tilly et al., 1995 and; Matsuda-Minehata et al., 2006). GSPs relieve the oxidative stress of testicular tissue caused by arsenic and cisplatin in rodents (Genç et al., 2014; Zhao et al., 2014; Li et al., 2015), skeletal muscle of diabetic rats induced by low-dose streptomycin and high-sugar/high-fat (Ding et al., 2013), cyclosporine A poisoning in the heart tissue (Ozkan et al., 2012) and the porcine ovary induced by diquat (Zhang et al., 2019). Meanwhile, GSPs pretreatment effectively alleviates the apoptosis of porcine GCs induced by oxidative stress *in vitro* (Zhang et al., 2019) and ovarian aging by increasing antioxidant enzyme activity and the expression of antioxidant genes in aging ovaries (Liu X. et al., 2018). Consistent with the previous report, GSPs improved T-AOC in H_2_O_2_-treated GCs from D280 chicken SYFs. Our studies revealed that GSPs enhanced the proliferation capacity and decreased the ROS level in H_2_O_2_-treated GCs from D280 chicken SYFs and SWFs from D580 chicken. Moreover, oxidative stress-activated autophagy has been reported to initiate programmed cell death without apoptosis induction in multiple types of mammalian cells (Choi et al., 2010). To better evaluate whether GSPs mediated autophagy inhibition or apoptosis, we observed the autophagic formation in GCs treated with/without ZVF (apoptosis inhibitors). The ZVF alone did not completely increase the cell viability of H_2_O_2_-treated GCs from D280 chicken SYFs, indicating a not only apoptosis in the process of aging.

Interestingly, GSPs have been recently proposed to inhibit autophagy through redox-mediated elimination of free radicals (Shi et al., 2019). Therefore, this study explored whether autophagy is related to the inhibitory effect of GSPs on GC oxidative damage. These results manifested that GSPs decreased autophagy to protect the ovaries from oxidative stress. GSPs decreased the up-regulation of autophagy related genes (*Rab7, Pik3c3, Map1lc3b, Atg7, Becn1, Bnip3, and Atg12*) induced by H_2_O_2_. We compared ovarian histology, follicle histology, and autophagy markers to investigate the effects of aging on ovary antioxidant status. Our results demonstrated that GSPs decreased the LC3B protein level and enhanced the SQSTM1 protein level, thus increasing the cell viability similar to the effect of 3-MA. However, pretreatment of GSPs did not eliminate the accumulation of ROS in H_2_O_2_-treated GCs. Collectively, this study first describes GSPs alleviate cellular oxidative stress by inhibiting autophagy in the aging process. We then considered the potential of GSPs to relieve autophagy in H_2_O_2_-induced aging ovaries. Meanwhile, GSPs supplementation effectively alleviated the oxidative stress in aging ovaries by reducing autophagy. To better evaluate GSPs mediated autophagy inhibition, we observed the autophagic formation in GCs treated with/without DC661 (lysosomal protease inhibitors). The DC661 alone did not significantly increase the accumulation of LC3B protein expression, indicating a low baseline autophagic formation in GCs under the normal growth process. Our data displayed that GSPs decreased the level of ROS and GSPs-mediated inhibition of autophagic GCs death is not just dependent on ROS clearance.

There are many factors affecting autophagy. Several studies have shown that there is a connection between SIRT1 and autophagy. In the study of oridonin inhibiting the proliferation of human cervical cancer HELA cells and myeloma RPMI8266 cells, it was found that autophagy was activated and the expression level of SIRT1 increased. After using 3-MA to inhibit autophagy, the level of SIRT1 also decreased (Cui et al., 2006; Zeng et al., 2012). Cardiomyocytes cultured *in vitro* can induce autophagy in a SIRT1 and FOXO1 dependent manner in the glucose-free medium (Mukherjee et al., 2010). The artificial regulation of the expression level of SIRT1 also further illustrates its role in autophagy. The silence of SIRT1 increased the sensitivity of neurons to prion-induced cell death and mitochondrial dysfunction (Morselli et al., 2010; Jeong et al., 2012). Although SIRT1 is not an essential part of the autophagy mechanism, autophagy induction requires activation of the SIRT1 signal transduction process or SIRT1 participation in regulation.

SIRT1 not only directly acted on the components of the autophagy mechanism to affect autophagy but also promoted or enhanced the expression of the components of the autophagy mechanism through its deacetylation of a series of transcription factors (Morselli et al., 2010; Jeong et al., 2012). The most well-known of these transcription factors are members of the FOXO family (Katoh and Katoh, 2004). FoxO1, one of the key downstream targets of the Akt pathway, is an important nuclear transcription factor that regulates diverse cellular responses involving cell differentiation, cellular metabolism, and the inflammatory response (Xu et al., 2020). The expression of FoxO1 was up-regulated by 3-NP intraperitoneal injection or H_2_O_2_ treatment and the mRNA expression of apoptosis-related genes and the apoptosis rate of granulosa cells also increased (Zhou et al., 2020). FoxO1 was selectively overexpressed in ovarian GCs (Liu et al., 2009), which has a potential role in regulating GC function (Kyung et al., 2018; Zhou et al., 2020). Meanwhile, it is a key factor in promoting GC death induced by oxidative stimulation (Park et al., 2005; Shen et al., 2014 and; Shen et al., 2017). Immunohistochemical analysis of rodent ovary showed that FoxO1 was concentrated in the nucleus of GCs in atresia follicles. In neuronal cells, oxidative stress causes FoxO1 to transfer from cytoplasm to nucleus, leading to cell death (Zheng et al., 2000). However, in our results, the FoxO1 protein was located in cell cytosolic, which was different from reported in the previous study (Shi et al., 2019).

Through these studies, we speculate that inhibition of FoxO1 may be the key to maintaining the health of GCs. However, it is unknown whether FoxO1 joins in the regulation of GCs protection during GSPs mediated oxidative damage. The functions of FoxO1 are strictly dominated by phosphorylation, acetylation, and ubiquitination (Tian et al., 2020). AC-FoxO1 is essential for gluconeogenesis regulation in the rapid feed cycle (Li et al., 2019). Importantly, ATG7 formed complex with AC-FoxO1, which was accompanied by intensified autophagic signals during oxidative stress. Interaction between ATG7 and AC-FoxO1 facilitates the autophagic activity (Tian et al., 2020). Meanwhile, the regulatory mode of AC-FoxO1 in the protective mechanism in GSPs has not been reported on chicken ovarian GCs. This study showed that GSPs significantly inhibited autophagy and oxidative damage of the follicular GCs through inhibition of AC-FoxO1 expression. In addition, the expression of AC-FoxO1 was increased in the H_2_O_2_-treated GCs and treatment of GSPs reduced the expression of AC-FoxO1 and enhanced the expression of SIRT1. Therefore, these results indicate that GSPs are capable of inhibiting autophagic GC death and follicular senescence in a FoxO1-dependent manner.

Taken together, the results from this study suggest that FoxO1 is a critical target of GSPs-mediated protection of GCs against aging. Autophagy is activated by FoxO1-inducing autophagic GC death under oxidative stress. GSPs inhibited FoxO1 transferring from cytoplasm into nuclei via activation of the PI3K-AKT pathway. Moreover, inhibition of FoxO1 acetylation through the SIRT1 pathway also attenuated autophagic death in GCs that was induced by H_2_O_2_. Therefore, GSPs maintain GCs survival against oxidative damage through inhibition of autophagy, downregulation of deacetylation of FoxO1 and SIRT1/FoxO1 transcriptional activity (see Graphical abstract).

## 5 Conclusion

In conclusion, increased autophagy occurs in ovaries of D580 aged hens compared with D280 young hens. Pretreatment of GSPs significantly restored the increased autophagy of H_2_O_2_-exposed GCs in culture and also from the naturally aging hens. GSPs relieved the decrease of GCs viability that was caused by oxidative stress-induced autophagy via suppressing FoxO1-dependent autophagy with activated PI3K-AKT-SIRT1-FoxO1 pathway. Therefore, targeting GSPs-FoxO1 signaling might provide a valuable measure of retarding ovarian aging in the laying chickens.

## Data Availability

The original contributions presented in the study are included in the article/Supplementary Material, further inquiries can be directed to the corresponding author.

## References

[B1] Agarwal A. Aponte-Mellado A. Premkumar B. J. Shaman A. Gupta S. (2012). The Effects of Oxidative Stress on Female Reproduction: A Review. Reprod. Biol. Endocrinol. 10, 49. 10.1186/1477-7827-10-49 22748101 PMC3527168

[B2] Alves-Fernandes D. K. Jasiulionis M. G. (2019). The Role of SIRT1 on DNA Damage Response and Epigenetic Alterations in Cancer. Int. J. Mol. Sci. 20 (13), 3153. 10.3390/ijms20133153 PMC6651129 31261609

[B3] Chen C. Zhou M. Ge Y. Wang X. (2020). SIRT1 and Aging Related Signaling Pathways. Mech. ageing Dev. 187, 111215. 10.1016/j.mad.2020.111215 32084459

[B4] Choi J. Y. Jo M. W. Lee E. Y. Yoon B.-K. Choi D. S. (2010). The Role of Autophagy in Follicular Development and Atresia in Rat Granulosa Cells. Fertil. Steril. 93 (8), 2532–2537. 10.1016/j.fertnstert.2009.11.021 20149359

[B5] Choi J. Jo M. Lee E. Choi D. (2011). Induction of Apoptotic Cell Death via Accumulation of Autophagosomes in Rat Granulosa Cells. Fertil. Sterility 95 (4), 1482–1486. 10.1016/j.fertnstert.2010.06.006 20630503

[B56] Cui Q. Tashiro S. Onodera S. Ikejima T. (2006). Augmentation of Oridonin-induced Apoptosis Observed with Reduced Autophagy. J. Pharmacol. Sci. 101 (3), 230–239. 10.1254/jphs.fpj06003x 16861822

[B6] Devine P. J. Perreault S. D. Luderer U. (2012). Roles of Reactive Oxygen Species and Antioxidants in Ovarian Toxicity1. Biol. Reprod. 86 (2), 27. 10.1095/biolreprod.111.095224 22034525 PMC3290661

[B7] Ding Y. Dai X. Jiang Y. Zhang Z. Bao L. Li Y. (2013). Grape Seed Proanthocyanidin Extracts Alleviate Oxidative Stress and ER Stress in Skeletal Muscle of Low-Dose Streptozotocin- and High-Carbohydrate/high-Fat Diet-Induced Diabetic Rats. Mol. Nutr. Food Res. 57 (2), 365–369. 10.1002/mnfr.201200463 23161660

[B8] Fu K. Chen L. Hu S. Guo Y. Zhang W. Bai Y. (2021). Grape Seed Proanthocyanidins Attenuate Apoptosis in Ischemic Stroke. Acta Neurol. Belg. 121 (2), 357–364. 10.1007/s13760-019-01111-9 30835051

[B9] Genç A. Üçok K. Şener Ü. Koyuncu T. Akar O. Çeli̇k S. (2014). Association Analyses of Oxidative Stress, Aerobic Capacity, Daily Physical Activity, and Body Composition Parameters in Patients with Mild to Moderate COPD. Turk. J. Med. Sci. 44 (6), 972–979. 10.3906/sag-1308-65 25552149

[B10] Jeong J. K. Moon M. H. Lee Y. J. Seol J. W. Park S. Y. (2012). Autophagy Induced by the Class III Histone Deacetylase Sirt1 Prevents Prion Peptide Neurotoxicity. Neurobiol. Aging 34 (1), 146–156. 10.1016/j.neurobiolaging.2012.04.002 22575359

[B11] Jinhwan L. Nakamura B. N. Isaac M. Kavanagh T. J. Ulrike L. (2015). Glutamate Cysteine Ligase Modifier Subunit (Gclm) Null Mice Have Increased Ovarian Oxidative Stress and Accelerated Age-Related Ovarian Failure. Endocrinology 156 (9), 3329–3343. 10.1210/en.2015-1206 26083875 PMC4541624

[B12] Katoh M. Katoh M. (2004). Human FOX Gene Family (Review). Int. J. Oncol. 25 (5), 1495–1500. 10.3892/ijo.25.5.1495 15492844

[B13] Kyung I. B. René R. Sevag P. Saffari A. Ma Z. Anh P. L. (2018). Ultrafine Particle Exposure Reveals the Importance of FOXO1/Notch Activation Complex for Vascular Regeneration. Antioxid. Redox Signal. 28 (13), 1209–1223. 10.1089/ars.2017.7166 29037123 PMC5912723

[B14] Li S. G. Ding Y. S. Niu Q. Xu S. Z. Pang L. J. Ma R. L. (2015). Grape Seed Proanthocyanidin Extract Alleviates Arsenic-Induced Oxidative Reproductive Toxicity in Male Mice. Biomed. Environ. Sci. 28 (4), 272–280. 10.3967/bes2015.038 25966753 PMC7135117

[B15] Li K. Qiu C. Sun P. Liu D. C. Wu T. J. Wang K. (2019). Ets1-Mediated Acetylation of FoxO1 Is Critical for Gluconeogenesis Regulation during Feed-Fast Cycles. Cell. Rep. 26 (11), 2998–3010.e5. 10.1016/j.celrep.2019.02.035 30865889

[B16] Li L. Geng X. Tian L. Wang D. Wang Q. (2020). Grape Seed Proanthocyanidins Protect Retinal Ganglion Cells by Inhibiting Oxidative Stress and Mitochondrial Alteration. Arch. Pharmacal Res. 43 (10), 1056–1066. 10.1007/s12272-020-01272-9 33078305

[B53] Lim J. Nakamura B. N. Mohar I. Kavanagh T. J. Luderer U. (2015). Glutamate Cysteine Ligase Modifier Subunit (Gclm) Null Mice Have Increased Ovarian Oxidative Stress and Accelerated Age-Related Ovarian Failure. Endocrinology 156 (9), 3329–3343. 10.1210/en.2015-1206 26083875 PMC4541624

[B17] Lin X. Liu X. Ma Y. Mi Y. Zhang C. (2018a). Coherent Apoptotic and Autophagic Activities Involved in Regression of Chicken Postovulatory Follicles. Aging 10 (4), 819–832. 10.18632/aging.101436 29706614 PMC5940126

[B18] Lin Y. Sheng M. Ding Y. Zhang N. Song Y. Du H. (2018b). Berberine Protects Renal Tubular Cells against Hypoxia/reoxygenation Injury via the Sirt1/p53 Pathway. J. Nat. Med. 72 (3), 715–723. 10.1007/s11418-018-1210-1 29680964

[B19] Liu Z. Rudd M. D. Inmaculata H. G. Ignacio G. R. Fan H. Y. Zeleznik A. J. (2009). FSH and FOXO1 Regulate Genes in the Sterol/steroid and Lipid Biosynthetic Pathways in Granulosa Cells. Mol. Endocrinol. 23 (5), 649–661. 10.1210/me.2008-0412 19196834 PMC2675958

[B20] Liu X. T. Lin X. Zhang S. Y. Guo C. Q. Li J. Mi Y. (2018a). Lycopene Ameliorates Oxidative Stress in the Aging Chicken Ovary via Activation of Nrf2/HO-1 Pathway. Aging (Albany NY) 10 (8), 2016–2036. 10.18632/aging.101526 30115814 PMC6128425

[B21] Liu X. T. Lin X. Mi Y. L. Li J. Zhang C. Q. (2018b). Grape Seed Proanthocyanidin Extract Prevents Ovarian Aging by Inhibiting Oxidative Stress in the Hens. Oxid. Med. Cel. Longev. 2018, 9390810. 10.1155/2018/9390810 PMC5818927 29541349

[B22] Lu G. D. Shen H. M. Chung M. C. Ong C. N. (2007). Critical Role of Oxidative Stress and Sustained JNK Activation in Aloe-Emodin-Mediated Apoptotic Cell Death in Human Hepatoma Cells. Carcinogenesis 28 (9), 1937–1945. 10.1093/carcin/bgm143 17698970

[B23] Luderer U. (2014). Ovarian Toxicity from Reactive Oxygen Species. Vitamins Horm. 94, 99. 10.1016/b978-0-12-800095-3.00004-3 24388188

[B24] Matsuda-Minehata F. Inoue N. Goto Y. Manabe N. (2006). The Regulation of Ovarian Granulosa Cell Death by Pro- and Anti-Apoptotic Molecules. J. Reprod. Dev. 52 (6), 695–705. 10.1262/jrd.18069 16926526

[B25] Ming S. Cao Y. Yi J. Wei Y. Liu H. (2018). Melatonin Protects Mouse Granulosa Cells against Oxidative Damage by Inhibiting FOXO1-Mediated Autophagy: Implication of an Antioxidation-independent Mechanism. Redox Biol. 18, 138–157. 10.1016/j.redox.2018.07.004 30014903 PMC6068202

[B26] Morselli E. Maiuri M. C. Markaki M. Megalou E. Pasparaki A. Palikaras K. (2010). The Life Span-Prolonging Effect of Sirtuin-1 Is Mediated by Autophagy. Autophagy 6 (1), 186–188. 10.4161/auto.6.1.10817 20023410

[B57] Mukherjee P. Winter S. L. Alexandrow M. G. (2010). Cell Cycle Arrest by Transforming Growth Factor Beta1 Near G1/S is Mediated by Acute Abrogation of Prereplication Complex Activation Involving an Rb-MCM Interaction. Mol. Cell. Biol. 30 (3), 845–856. 10.1128/MCB.01152-09 19948884 PMC2812244

[B27] Nicole D. Olga Z. Marcin N. Albert R. Hmeidan F. A. Verona B. (2006). Lectin-Like Oxidized Low-Density Lipoprotein Receptor-1-Mediated Autophagy in Human Granulosa Cells as an Alternative of Programmed Cell Death. Endocrinology 147 (8), 3851–3860. 10.1210/en.2006-0088 16690797

[B28] Orrenius S. Gogvadze V. Zhivotovsky B. (2017). Mitochondrial Oxidative Stress: Implications for Cell Death. Annu. Rev. Pharmacol. Toxicol. 47, 143–183. 10.1146/annurev.pharmtox.47.120505.105122 17029566

[B29] Ozkan G. Ulusoy S. Alkanat M. Orem A. Akcan B. Ersöz S. (2012). Antiapoptotic and Antioxidant Effects of GSPE in Preventing Cyclosporine A-Induced Cardiotoxicity. Ren. Fail. 34 (4), 460–466. 10.3109/0886022X.2012.656563 22299713

[B30] Park Y. Maizels E. T. Feiger Z. J. Alam H. Peters C. A. Woodruff T. K. (2005). Induction of Cyclin D2 in Rat Granulosa Cells Requires FSH-dependent Relief from FOXO1 Repression Coupled with Positive Signals from Smad. J. Biol. Chem. 280 (10), 9135–9148. 10.1074/jbc.M409486200 15613482 PMC1564190

[B31] Rigotti M. Cerbaro A. F. da Silva I. Agostini F. Branco C. S. Moura S. (2020). Grape Seed Proanthocyanidins Prevent H2 O2 -induced Mitochondrial Dysfunction and Apoptosis via SIRT 1 Activation in Embryonic Kidney Cells. J. Food Biochem. 44 (3), e13147. 10.1111/jfbc.13147 31943241

[B32] Rodrigues B. L. C. Lallo M. A. Perez E. C. (2020). The Controversial Role of Autophagy in Tumor Development: A Systematic Review. Immunol. Invest. 49 (4), 386–396. 10.1080/08820139.2019.1682600 31726897

[B33] Serke H. Vilser C. Nowicki M. Hmeidan F. A. Blumenauer V. Hummitzsch K. (2009). Granulosa Cell Subtypes Respond by Autophagy or Cell Death to oxLDL-Dependent Activation of the Oxidized Lipoprotein Receptor 1 and Toll-like 4 Receptor. Autophagy 5 (7), 991–1003. 10.4161/auto.5.7.9507 19730000

[B55] Shen M. Lin F. Zhang J. Tang Y. Chen W. K. Liu H. (2012). Involvement of the Up-regulated FoxO1 Expression in Follicular Granulosa Cell Apoptosis Induced by Oxidative Stress. J. Biol. Chem. 287 (31), 25727–25740. 10.1074/jbc.M112.349902 22669940 PMC3406661

[B34] Shen M. Liu Z. Li B. Teng Y. Zhang J. Tang Y. (2014). Involvement of FoxO1 in the Effects of Follicle-Stimulating Hormone on Inhibition of Apoptosis in Mouse Granulosa Cells. Cell. Death Dis. 5 (10), e1475. 10.1038/cddis.2014.400 25321482 PMC4237239

[B35] Shen M. Jiang Y. Guan Z. Cao Y. Li L. Liu H. (2017). Protective Mechanism of FSH against Oxidative Damage in Mouse Ovarian Granulosa Cells by Repressing Autophagy. Autophagy 13 (8), 1364–1385. 10.1080/15548627.2017.1327941 28598230 PMC5584866

[B36] Shi Y. Jia M. Xu L. Fang Z. Wu W. Zhang Q. (2019). MiR‐96 and Autophagy Are Involved in the Beneficial Effect of Grape Seed Proanthocyanidins against High‐fat‐diet‐induced Dyslipidemia in Mice. Phytother. Res. 33 (4), 1222–1232. 10.1002/ptr.6318 30848548

[B37] Shi F. (2003). Relationship between FoxO1 Protein Levels and Follicular Development, Atresia, and Luteinization in the Rat Ovary. J. Endocrinol. 179 (2), 195–203. 10.1677/joe.0.1790195 14596671

[B38] Tan Y. Q. Zhang J. Zhou G. (2016). Autophagy and its Implication in Human Oral Diseases. Autophagy 13 (2), 225–236. 10.1080/15548627.2016.1234563 27764582 PMC5324841

[B39] Tatone C. Amicarelli F. Carbone M. C. Monteleone P. Caserta D. Marci R. (2008). Cellular and Molecular Aspects of Ovarian Follicle Ageing. Hum. Reprod. Update 14 (2), 131–142. 10.1093/humupd/dmm048 18239135

[B40] Tian Y. N. Chen H. D. Tian C. Q. Wang Y. Q. Miao Z. H. (2020). Polymerase Independent Repression of FoxO1 Transcription by Sequence-specific PARP1 Binding to FoxO1 Promoter. Cell Death Dis. 11 (1), 71. 10.1038/s41419-020-2265-y 31992690 PMC6987093

[B41] Tilly J. L. Tilly K. I. Kenton M. L. Johnson A. L. (1995). Expression of Members of the Bcl-2 Gene Family in the Immature Rat Ovary: Equine Chorionic Gonadotropin-Mediated Inhibition of Granulosa Cell Apoptosis Is Associated with Decreased Bax and Constitutive Bcl-2 and Bcl-Xlong Messenger Ribonucleic Acid Levels. Endocrinology 136 (1), 232–241. 10.1210/endo.136.1.7828536 7828536

[B42] Wang M. L. Suo X. Gu J. H. Zhang W. W. Fang Q. Wang X. (2008). Influence of Grape Seed Proanthocyanidin Extract in Broiler Chickens: Effect on Chicken Coccidiosis and Antioxidant Status. Poult. Sci. 87 (11), 2273–2280. 10.3382/ps.2008-00077 18931178

[B54] Wang X. Fu Y. F. Liu X. Feng G. Xiong D. Mu G. F. (2018). ROS Promote Ox-LDL-Induced Platelet Activation by Up-Regulating Autophagy Through the Inhibition of the PI3K/AKT/mTOR Pathway. Cell. Physiol. Biochem. 50 (5), 1779–1793. 10.1159/000494795 30384368

[B43] Xu J. Liu F. Xiong Z. Huo J. Li W. Jiang B. (2020). The Cleft Palate Candidate Gene BAG6 Supports FoxO1 Acetylation Topromote FasL-Mediated Apoptosis during Palate Fusion. Exp. Cel Res. 396 (2), 112310. 10.1016/j.yexcr.2020.112310, 32991875

[B44] Yao H. Zhen Y. Zhang S. Zhang W. Wen Z. (2018). Upregulation of Sirt1 Inhibits H_2_O_2_-Induced Osteoblast Apoptosis via FoxO1/β-Catenin Pathway. Mol. Med. Rep. 17 (5), 6681–6690. 10.3892/mmr.2018.8657 29512706

[B45] Yao J. Ma Y. Zhou S. Bao T. Mi Y. Zeng W. (2020). Metformin Prevents Follicular Atresia in Aging Laying Chickens through Activation of PI3K/AKT and Calcium Signaling Pathways. Oxid. Med. Cel. Longev. 2020, 3648040. 10.1155/2020/3648040 PMC7718058 33294120

[B46] Zeng R. He J. Peng J. Chen Y. Yi S. Zhao F. (2012). The Time-dependent Autophagy Protects against Apoptosis with Possible Involvement of Sirt1 Protein in Multiple Myeloma under Nutrient Depletion. Ann. Hematol. 91 (3), 407–417. 10.1007/s00277-011-1315-z 21915620

[B47] Zhang M. Zhang Q. Hu Y. Xu L. Jiang Y. Zhang C. (2017). miR-181a Increases FoxO1 Acetylation and Promotes Granulosa Cell Apoptosis via SIRT1 Downregulation. Cel Death Dis. 8 (10), e3088. 10.1038/cddis.2017.467 PMC5680589 28981116

[B48] Zhang J. Q. Wang X. W. Chen J. F. Ren Q. L. Wang J. Gao B. W. (2019). Grape Seed Procyanidin B2 Protects Porcine Ovarian Granulosa Cells against Oxidative Stress-Induced Apoptosis by Upregulating Let-7a Expression. Oxid. Med. Cel. Longev. 2019, 1076512. 10.1155/2019/1076512 PMC6885843 31827667

[B49] Zhao Y. M. Gao L. P. Zhang H. L. Guo J. X. Guo P. P. (2014). Grape Seed Proanthocyanidin Extract Prevents DDP-Induced Testicular Toxicity in Rats. Food Funct. 5 (3), 605–611. 10.1039/c3fo60486a 24504493

[B50] Zheng W. H. Kar S. Quirion R. (2000). Insulin-like Growth Factor-1-Induced Phosphorylation of the Forkhead Family Transcription Factor FKHRL1 Is Mediated by Akt Kinase in PC12 Cells. J. Biol. Chem. 275 (50), 39152–39158. 10.1074/jbc.M002417200 10995739

[B51] Zhou L. Ding X. Wang J. Bai S. Zhang K. (2020). Oxidized Oils and Oxidized Proteins Induce Apoptosis in Granulosa Cells by Increasing Oxidative Stress in Ovaries of Laying Hens. Oxid. Med. Cel. Longev. 2020 (5), 1–11. 10.1155/2020/2685310 PMC7422066 32831991

[B52] Zhou S. Ma Y. Yao J. Zhao A. Zhang C. (2021). TGF-β1-induced Collagen Promotes Chicken Ovarian Follicle Development via an Intercellular Cooperative Pattern. Cell. Biol. Int. 45 (6), 1336–1348. 10.1002/cbin.11580 33675281

