# Supplementary figures and images for: Protective Effect of Grape Seed Proanthocyanidins on Oxidative Damage of Chicken Follicular Granulosa Cells by Inhibiting FoxO1-Mediated Autophagy
Source: Front Cell Dev Biol. 2022 Feb 15;10:762228. doi: 10.3389/fcell.2022.762228 (PMC8886245; doi:10.3389/fcell.2022.762228)

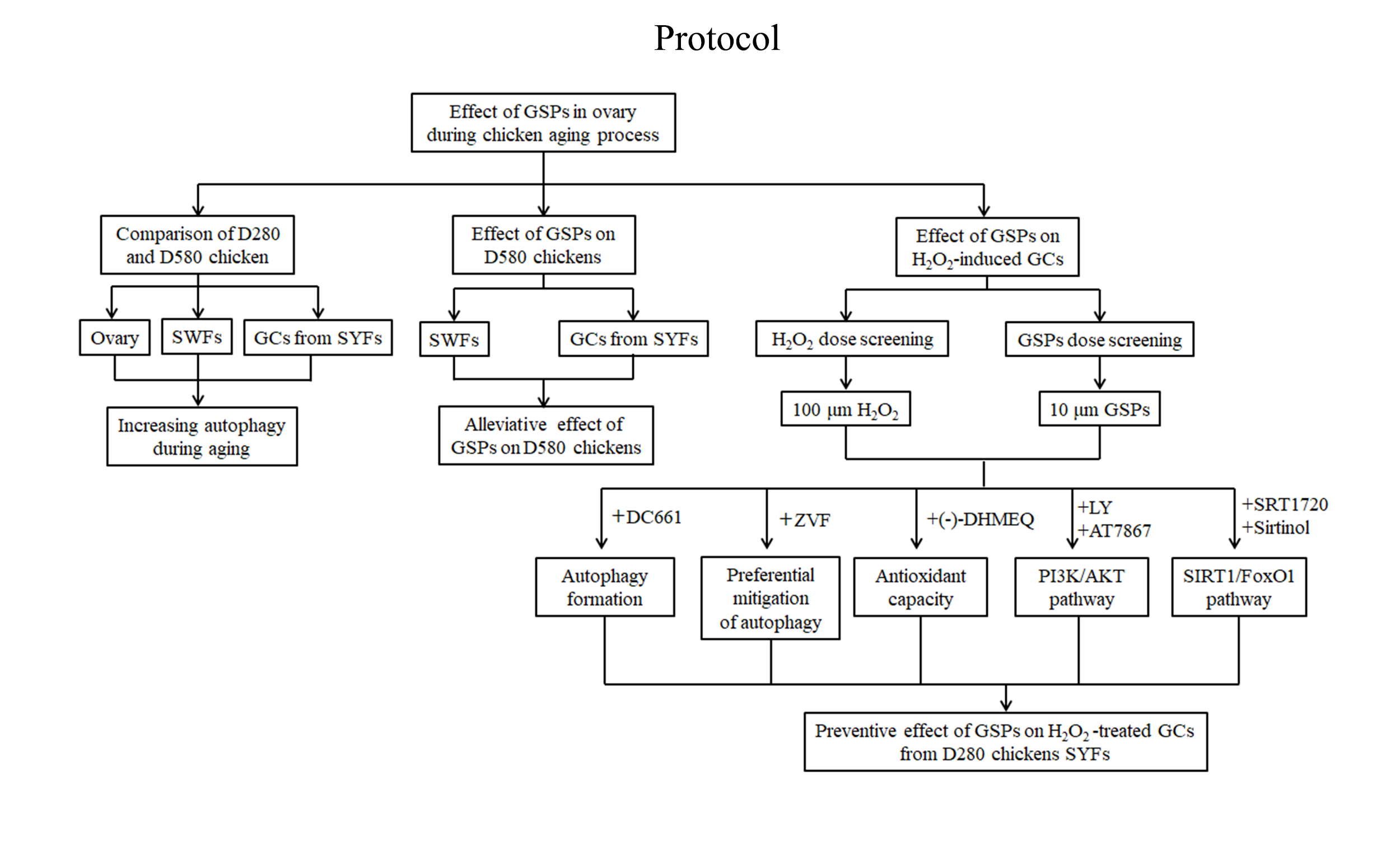

Supplement: Supplementary file 1 [file Image1.tif]
